# MicroRNA-195 prevents hippocampal microglial/macrophage polarization towards the M1 phenotype induced by chronic brain hypoperfusion through regulating CX3CL1/CX3CR1 signaling

**DOI:** 10.1186/s12974-020-01919-w

**Published:** 2020-08-20

**Authors:** Meng Mao, Yi Xu, Xin-Yu Zhang, Lin Yang, Xiao-bin An, Yang Qu, Ya-ni Chai, Yan-Ru Wang, Ting-ting Li, Jing Ai

**Affiliations:** Department of Pharmacology (The State-Province Key Laboratories of Biomedicine-Pharmaceutics of China), College of Pharmacy of Harbin Medical University, Harbin, 150086 Heilongjiang Province China

**Keywords:** Chronic brain hypoperfusion, MicroRNA-195, Microglial/macrophage polarization, Neuronal-microglial cross-talk, Neuroinflammation

## Abstract

**Background:**

Microglial polarization is a dynamic response to acute brain hypoxia induced by stroke and traumatic brain injury (TBI). However, studies on the polarization of microglia in chronic cerebral circulation insufficiency (CCCI) are limited. Our objective was to investigate the effect of CCCI on microglial polarization after chronic brain hypoperfusion (CBH) and explore the underlying molecular mechanisms.

**Methods:**

CBH model was established by bilateral common carotid artery occlusion (2-vessel occlusion, 2VO) in rats. Using the stereotaxic injection technique, lenti-pre-*miR-195* and anti-*miR-195* oligonucleotide fragments (lenti-pre-AMO-*miR-195*) were injeted into the CA1 region of the hippocampus to construct animal models with high or low expression of *miR-195*. Immunofluorescence staining and flow cytometry were conducted to examine the status of microglial polarization. In vitro, Transwell co-culture system was taken to investigate the role of *miR-195* on neuronal-microglial communication through CX3CL1-CX3CR1 signaling. Quantitative real-time PCR was used to detect the level of *miR-195* and inflammatory factors. The protein levels of CX3CL1 and CX3CR1 were evaluated by both western blot and immunofluorescence staining.

**Results:**

CBH induced by 2VO initiated microglial/macrophage activation in the rat hippocampus from 1 week to 8 weeks, as evaluated by increased ratio of (CD68^+^ and CD206^+^)/Iba-1 immunofluorescence. And the microglial/macrophage polarization was shifted towards the M1 phenotype at 8 weeks following CBH. The expression of CX3CL1 and CX3CR1 was increased in the hippocampus of 2VO rats at 8 weeks. An in vitro study in a Transwell co-culture system demonstrated that transfection of either primary-cultured neonatal rat neurons (NRNs) or microglial BV2 cells with AMO-195-induced M1 polarization of BV2 cells and increased CX3CL1 and CX3CR1 expression and that these effects were reversed by *miR-195* mimics. Furthermore, the upregulation of *miR-195* induced by lenti-pre-*miR-195* injection prevented microglial/macrophage polarization to M1 phenotype triggered by hippocampal injection of lenti-pre-AMO-*miR-195* and 2VO surgery.

**Conclusions:**

Our findings conclude that downregulation of *miR-195* in the hippocampus is involved in CBH-induced microglial/macrophage polarization towards M1 phenotype by governing communication between neurons and microglia through the regulation of CX3CL1 and CX3CR1 signaling. This indicates that *miR-195* may provide a new strategy for clinical prevention and treatment of CBH.

## Background

Microglia play an important role in various neurodegenerative diseases, including Alzheimer’s disease (AD), Parkinson’s disease (PD), Huntington’s disease (HD), and prion diseases [1, 2]. Chronic brain hypoperfusion (CBH), one of the major pre-clinical phases of AD [3, 4], has been found to cause extracellular Aβ aggregation [5], tau protein hyperphosphorylation [6], neuronal loss [7], and even early astrocytic glial activation [8]. However, the influence of CBH on microglial function is unclear.

Microglia are immune cells that are considered the brain’s first line of defense and are double-edged swords in responding to pathogens and supporting CNS homeostasis and plasticity [9, 10]. It has been documented that resident microglia and peripheral macrophages act as guardians to respond to various types of acute brain injury via activation [11–13]. Although growing evidences demonstrated activated microglia and/or macrophages may include a spectrum but functionally overlapping phenotypes after injury [13–15], the activated microglia/macrophages can be roughly divided into two different states according to their functions [16, 17]. The first is classically activated M1 microglia/macrophage, which release proinflammatory mediators, while the second is alternatively activated M2 microglia/macrophage, which release numerous protective/trophic factors [15, 18]. Acute severe brain ischaemia, such as intracerebral hemorrhage (ICH) and traumatic brain injury (TBI), has been found to be associated with an early M2 phenotype, followed by a transition to the M1 phenotype [14, 19, 20]. However, whether CBH also induces dynamic polarization of microglial/macrophage in the hippocampus is unknown.

Chemokine (C-X3-C motif) ligand 1 (CX3CL1), which is anchored to the neuronal membrane, is an intriguing chemokine that plays a central role in microglial activation by interacting with CX3CR1 expressed by microglia cells [21, 22]. Interestingly, clinical studies have reported increased levels of CX3CL1 in patients with moderate AD, while significantly decreased levels of CX3CL1 in patients with advanced AD [23, 24]. In addition, CX3CR1 deficiency results in fewer apoptotic neurons, reduced ROS levels, facilitated alternative activation (towards the M2 phenotype) of microglia/macrophages, and attenuated synthesis and release of inflammatory cytokines in a CX3CR1^−/−^ MCAO mouse model [25] and the number of microglia surrounding Aβ deposits in a mouse model of AD [26]. However, whether the CX3CL1/CX3CR1 pathway dysfunction is also involved in CBH pathology process, a kind of chronic mild ischaemia, remains unclear.

It has been reported that *miR-195* protects against focal acute cerebral ischemia-induced cell apoptosis by targeting CX3CR1 [27]. Additionally, *miR-195* reduces the expression of multiple NF-κB downstream effectors by directly targeting IKKα and TAB3 in hepatocellular carcinoma (HCC) [28] and inhibits an M1-like polarization-induced proinflammatory profile in macrophages [29]. Nevertheless, whether *miR-195* participated in CBH-induced hippocampal microglial/macrophage polarization by regulating CX3CL1-CX3CR1 signaling pathway has not been studied. Therefore, the purpose of this study was to investigate the effect of CBH on microglial/macrophage polarization and to explore the potential molecular mechanisms of *miR-195* on this process.

## Materials and methods

### Experimental design

The animal groups and number of rats used in the study are listed in Supplementary Table S1, Additional file 1.

To investigate how CBH affects microglial/macrophage activation, we detected the polarization phenotypes of microglia/macrophage in the hippocampus of rats after 2VO surgery by immunofluorescence staining, flow cytometry analysis, and qRT-PCR. Then, to investigate the role of *miR-195* on hippocampal microglial/macrophage polarization in rats following CBH, we designed lentiviral constructs, named lenti-pre-*miR-195* and lenti-AMO- *miR-195*, and stereotaxically injected them directly into the bilateral hippocampal CA1 subfields of each rat. We evaluated the polarization phenotypes of microglial/macrophage and related proteins in the hippocampus of these rats by immunofluorescence staining, qRT-PCR, and western blotting. The potential molecular mechanisms of *miR-195* on hippocampal microglial polarization were examined in vitro Transwell co-culture system.

### Animals

A total of 99 adult male Sprague Dawley (SD) rats used in the study (280–300 g) were supplied by Changsheng Biotechnology (Liaoning, Shenyang Province, China). All animals were housed at 23 ± 1 °C with 55 ± 5% humidity and maintained on a 12-h artificial dark-light cycle (lights on at 7:00 AM) with food and water available ad libitum. All animal handling and surgical procedures were performed in accordance with the guidelines established by the National Institutes of Health for the care and use of laboratory animals and were approved by the Institutional Animal Care and Use Committee at Harbin Medical University (China).

### Permanent bilateral occlusion of the common carotid arteries (2VO) in rats

Bilateral common carotid artery occlusion (2-vessel occlusion, 2VO) in rats was prepared according to our previous studies [1, 30]. In brief, animals were anesthetized with chloral hydrate (300 mg/kg). No. 3–0 silk sutures were placed under the separated common carotid artery and tightened up. Then, the common carotid artery was cut off between the two ligated silk sutures. The same procedure was performed on the sham group but without the ligation. The brain tissues and slices were harvested from 2VO rats at the time points of 1 week, 2 weeks, 4 weeks, and 8 weeks for the subsequent experiments.

### Primary culture of neonatal rat hippocampal neurons (NRNs)

Primary neuron cultures were prepared as previously described [31]. In brief, the hippocampus were removed from postnatal day 0 (P0) male SD rat pups and dissociated cells were maintained in DMEM containing 10% fetal bovine serum (FBS, HyClone, Logan, UT, USA) and subsequently inoculated into a poly-d-lysine precoated 6-well plate at a density of 1–2 × 10^6^ cells/well. After 4 h of incubation, the culture medium was replaced with neurobasal medium (Gibco, USA) with 2% B27 supplement (Invitrogen, USA). Cultures were placed in a 37 °C humidified atmosphere with 5% CO_2_. Neurons were collected from days 5–7 of in vitro culture were used for the experiments.

### Cell culture of BV2 cells

BV2 microglial cells were purchased from the National Infrastructure of Cell Line Resource and were seeded at a density of 1–2 × 10^5^ cells/well on 6-well plate format in DMEM (Invitrogen, USA) supplemented with 0.1% penicillin-streptomycin (Solarbio, China) and 10% fetal bovine serum (FBS, HyClone, Logan, UT, USA). Cultures were maintained at 37 °C in a 5% CO_2_ humidified atmosphere for 3 days, which were then collected for further experiments.

### Synthesis of oligonucleotides and cell transfection

*MiR-195* mimics, AMO-195, and NC were synthesized by the GenePharma Corporation (Suzhou, China) as previously described [7]. Cx3cl1-masking antisense ODNs were synthesized by the Sangon Biotech Corporation (Shanghai, China). The sequence of Cx3cl1-ODN, which was used to mask the binding sites of *miR-195* located in the 240-246 bp region of the *Cx3cl1* 3′UTR, was 5′—+C+C+A+G+CCAGCAGCAGAG+G+A+U+U+C—3′. The sequence of Cx3cr1-ODN, which was used to mask the binding sites of *miR-195* located in the 1236-1242 bp region in the *Cx3cr1* 3′UTR, was 5′—+C+C+A+C+GCAGCAGCACCU+G+C+A+G+G+C—3′. The nucleotides or deoxynucleotides at both ends of the antisense molecules were locked by a methylene bridge connecting the 2′-O and the 4′-C atoms. These plasmids were transfected into cells using X-treme GENE siRNA transfection reagent (Roche, Switzerland) according to the manufacturer’s instructions. Forty-eight hours after transfection, the cells were processed for further experiments.

### Co-culture of BV2 cells with neurons

After neonatal rat neurons (NRNs) were transfected with *miR-195*, AMO-195, and Cx3cl1-ODN for 48 h, the culture medium was discarded, and the NRNs were gently washed 3 times with PBS. Then, the NRNs were removed from the 6-well plates and placed on the bottom of a Transwell plate (Corning Company, USA). After washing 3 times with PBS, the cultured BV2 cells were placed in the upper chamber of the Transwell plate, which was separated from the NRNs by a semipermeable membrane (pore size of 0.4 μm). DMEM supplemented with 10% FBS was placed in the two separate chambers, and the plate was placed in an incubator at 37 °C with saturated humidity and 5% CO_2_. After 24 h of co-culture, both BV2 cells and neurons were collected for further experiments [31].

### Construction of lentivirus vectors

The details of the construction of the lentivirus vectors were described in our previous studies [5, 7]. The synthesis and lentiviral packaging of two double-stranded oligonucleotides, pre-*miR-195* and NC, and a single-stranded DNA oligonucleotide, pre-AMO-*miR-195*, were performed by the GeneCopoeia Inc. (Rockville, MD, USA).

### Stereotaxic injection of lentiviral vectors

Rats were anesthetized with chloral hydrate (300 mg/kg) and placed in an animal stereotaxic apparatus (RWB Life Science Co, Ltd., China). The injection coordinates relative to bregma were as follows: anteroposterior, − 4.52 mm; mediolateral, ± 3.2 mm; dorsoventral, − 3.16 mm below the surface. The coordinates were determined based on the atlas by Paxinos and Watson. A total of 2 μL (10,000 TU/μL) lenti-pre-*miR-195* and/or lenti-pre-AMO-*miR-195* was injected into the CA1 region of the hippocampus using a 5-μl Hamilton syringe with a 33-gauge needle (Hamilton, Bonaduz, Switzerland). Subsequent experiments were performed 8 weeks after virus injection [5, 7].

### Immunofluorescence staining

Rats were anesthetized and perfused with PBS (0.01 M, pH 7.40, 4 °C) transcardially first and then perfused with 4% paraformaldehyde (PFA) solution (pH 7.40, 4 °C). The brains were dissected in 4% PFA solution for fixation (24 h, 4 °C). The samples were transferred to a 30% sucrose solution for dehydration (72 h, 4 °C). After that, the brain samples were frozen (− 80 °C). Frozen brain tissues were then sliced into coronal slices (20-μm thick) in a cryostat microtome (Leica Microsystems, Germany), mounted on APS (Amino Silane)-coated glass slide (Solarbio, China), and stored in a − 80 °C freezer until analysis. Before staining, the 20-um-thick slices were incubated in PBS containing 0.3% Triton X-100 and 10% goat serum for 2 h at room temperature. After blocking, the slices were incubated with an anti-Iba-1 (1:500, Cat. #019-19741, Wako, USA), anti-rat-CD68 (1:200, Cat. #MCA341R, Bio-Rad, UK), or anti-rat-CD206 (1:200, Cat. #sc-58986, Santa Cruz, USA) primary antibody overnight at 4 °C followed by secondary antibodies conjugated to Alexa Fluor 488 (1:300, Cat. #A-21206, Invitrogen, USA) and Alexa Fluor 594 (1:300, Cat. #A-21203, Invitrogen, USA) and DAPI (1:300, Cat. #C1002, Beyotime, China) the next day.

The cultured cells were fixed in 4% PFA for 30 min and then incubated with PBS containing 0.1%Triton X-100 and 10% goat serum for 1 h at room temperature. After blocking, they were incubated with a rat anti-CX3CL1 primary antibody (1:300, Cat. #AF537, R&D system, USA) to detect neurons and an anti-Iba-1 (1:500, Cat. #019-19741,Wako, USA), anti-mouse CD68 primary antibody (1:300, Cat. #MCA1957, AbDserotec, Oxford, UK), or anti-mouse CD206 primary antibody (1:300, Cat. #MCA2235, AbDserotec, Oxford, UK) to detect BV2 cells overnight at 4 °C followed by secondary antibodies conjugated to Alexa Fluor 488 (1:500, Cat. #A-21206, Invitrogen, USA) and Alexa Fluor 594 (1:500, Cat. #A-21203, Invitrogen, USA) and DAPI (1:300, Cat. #C1002, Beyotime, China) the next day.

The fluorescence signals were detected using a fluorescence microscope (Zeiss Axio Scope A1) using × 20 objective with the same condition at a resolution of 1024 × 1024 pixels (24 bit). The size of the analyzed region is 232.45 μm × 232.45 μm. The number of cells in the dentate gyrus (DG) or CA1 region of the hippocampus in each animal was calculated by ImageJ software (NIH, MD, USA). Iba-1^+^ cells, CD68^+^/Iba-1^+^, and CD206^+^/Iba-1^+^ cells were counted in a blinded manner. The mean values were calculated from 3 randomly selected microscopic fields from each section for each animal. A total of 3 animals per group were analyzed. The data are expressed as the mean number of cells per square millimeter. Postprocessing of the images were limited to contrast adjustment without color adjustment.

### Isolation and purification of microglial cells

After anesthetization with chloral hydrate (300 mg/kg), the rats were transcardially perfused with PBS (0.01 M, pH 7.40, 4 °C). Microglial cells were extracted and isolated from the hippocampus according to a protocol published on bio-protocol [32]. Briefly, the homogenized tissues were digested to form cell suspensions, which were then filtered through a 70-μm nylon filter and then centrifuged at 300×*g* for 10 min. The cell pellets were resuspended in 20 ml of 30% isotonic Percoll solution (GE Healthcare, Uppsala, Sweden). Then, we transferred 10 ml of the resuspended cell pellets to a 50-ml centrifugation tube and carefully added 10 ml of 80% isotonic Percoll solution to the bottom of the centrifugation tube using a serological pipette with the help of gravity. Finally, the rest of the cell pellet was gradually added to the upper layer of the centrifugation tube, which was then centrifuged at 1050×*g* for 40 min at room temperature. The microglia were then collected from the interphase between the 80% and 30% Percoll layers. The cells were washed and resuspended in sterile HBSS and used for flow cytometry.

### Flow cytometry analysis of immunostained cells

Flow cytometry analysis of immunostained cells was performed following standard cell protocols. Prior to antibody labelling, the cell suspensions were incubated with anti-murine CD16/CD32 FC-Receptor (1:100, Cat. #14-0161-81, eBioscience, CA, USA) blocking reagent at 4 °C for 10 min. After blocking, the microglia were stained with FITC-conjugated mouse anti-rat CD11b (1:100, Cat. #554982, BD Biosciences, USA) and PerCP/Cy5.5-conjugated anti-rat CD45 (1:50, Cat. #202220, Biolegend, CA, USA). The microglia were then fixed and permeabilized with BD fixation/permeabilization solution (Cat. #554714, BD Cytofix/Cytoperm™, USA) for 20 min. The microglia were washed with BD Perm/Wash buffer (Cat. #554714, BD Cytofix/Cytoperm™, USA), resuspended in BD Perm/Wash buffer, and incubated with anti-iNOS (1:100, Cat. #ab15323, Abcam, UK) and rabbit mAb anti-arginase-1 (Arg-1) (1:100, Cat. # 3668s, Cell Signaling Technology, USA) primary antibodies for 30 min followed by a PE-conjugated anti-rabbit IgG (H+L) secondary antibody (1:100, Cat. #8885s, Cell Signaling Technology, USA). The cells were analyzed using a CytoFLEX instrument (Beckman Coulter Biotechnology, SuZhou). Ten thousand events were recorded, and microglia were identified by CD11b^+^/CD45^low^ expression [33]. The results were analyzed using CytExpert software (Beckman Coulter Biotechnology, SuZhou).

### Western blot analysis

Total protein was extracted from the hippocampus of rats or primary cultured neurons for immunoblotting analysis. The protein concentrations of all extracted samples were measured using the Bio-Rad Protein Assay (BioRad, Hercules, CA) with bovine serum albumin (BSA) standards. Fifty micrograms of the protein samples was separated by SDS-PAGE and then transferred to PVDF membranes, which were then incubated with primary antibodies at 4 °C overnight followed by fluorescent secondary antibodies (LICOR Biosciences, Lincoln, NE, USA). Anti-CX3CL1 (1:1000, Cat. #ab25088, Abcam, USA) and anti-CX3CR1 (1:1000, Cat. #ab8021, Abcam, USA) were used as the primary antibodies. β-Actin (1:1000, Cat. #G8795, Sigma, St. Louis, MO, USA) was used as an internal control. The bands on the blot were detected with the Odyssey infrared imaging system (LICOR Biosciences, Lincoln, USA). The signal intensities were analyzed using Odyssey v. 1.2 software and normalized to the intensity of the loading control, β-actin.

### Real-time PCR

Total RNA was extracted from the rat hippocampus or neurons using TRIzol (Invitrogen, USA) according to the manufacturer’s protocol. RNA was reverse transcribed into cDNA using a ReverTra AceqPCR RT Kit (Toboyo Co., Osaka, Japan). qPCR reactions were run in a volume of 20 μl using FastStart Universal SYBR Green Master (Roche, Switzerland) in an Applied Biosystems machine (Thermo Fisher Scientific, USA). The protocol was (1) 10 min at 95 °C, (2) 15 s at 95 °C, 30 s at 60 °C, and 30 s at 72 °C for 40 cycles and (3) melt curve analysis. The qPCR primer sequences were as follows: *miR-195:* forward (F), GGGGTAGCAGCACAGAAAT and reverse (R), TCCAGTGCGTGTCGTGGA; *U6*: F, GCTTCGGCAGCACATATACTAAAAT and R, CGCTTCACGAATTTGCGTGTCAT; *IL-1β*: F, GGCAACTGTCCCTGAACT and R, TCCACAGCCACAATGAGT; *TNF-α*: F, GACCCTCACACTCAGATCATCTTCT and R, TGCTACGACGTGGGCTACG; *TGF-β*: F, TGGCCAGATCCTGTCCAAAC and R, GTTGTACAAAGCGAGCACCG; and *GAPDH*: F, CTGGCATTGCTCTCAATGACAAC and R, CTTGCTCAGTTTATCCGCTGGCTG. The results were normalized against the internal control using the δ–δ CT method [5].

### Statistical analysis

The data are presented as the mean ± SD. Student’s *t* test was used for statistical analysis of differences between two groups. Each data set was analyzed for its ability to meet the statistical assumptions for equality of the variance, for normal distribution, and for sphericity. Independent sample test was calculated using the Levene variance equality test. If *P* > 0.05, independent student’s *t* test was used for the comparison between two groups. If *P* < 0.05 Kruskal-Wallis rank sum test was performed. One-way ANOVA was performed for the comparison among multivariate groups, and post hoc analyses of the significant main effect were further examined using Tukey tests. Graphs were generated using GraphPad Prism 8.0 software (La Jolla, CA, USA).

## Results

### CBH induces microglial/macrophage activation and polarization in the rat hippocampus

**Fig. 1:**
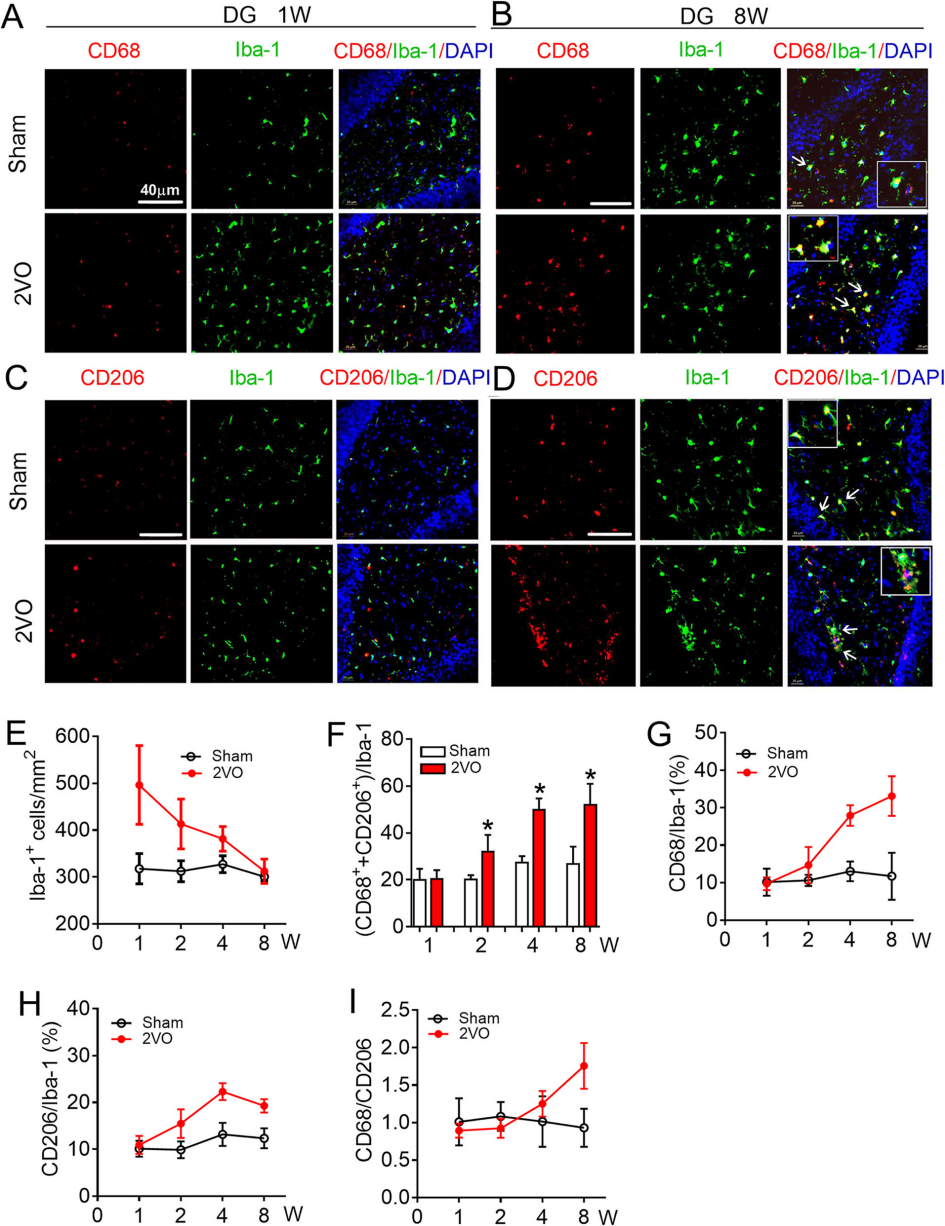
Microglial/macrophage activation and polarization in the dentate gyrus (DG) region of 2VO rats. **a**, **b** Representative images of CD68 expression in Iba-1^+^cells in hippocampal DG region of sham and 2VO rats at 1 week (a) and 8 weeks (**b**). **c**, **d** Representative images of CD206 expression in Iba-1^+^cells in hippocampal DG region of sham and 2VO rats at 1 week (**c**) and 8 weeks (**d**) by immunofluorescence staining. Scale: 40 μm. **e** Quantification of the Iba-1^+^ cell number in hippocampal DG region. **f** Quantification of the total polarized microglia/macrophage number in hippocampal DG region. **g** Quantification of the ratio of CD68 in Iba-1^+^cells in hippocampal DG region. **h** Quantification of the ratio of CD206 in Iba-1^+^ cells in hippocampal DG region.**i** Quantification of the ratio of CD68 and CD206 in hippocampal DG region. Values are mean ± SD, *n* = 9 slices from 3 animals per group, *P < 0.05 vs sham group, Student’s *t* test

**Fig. 2:**
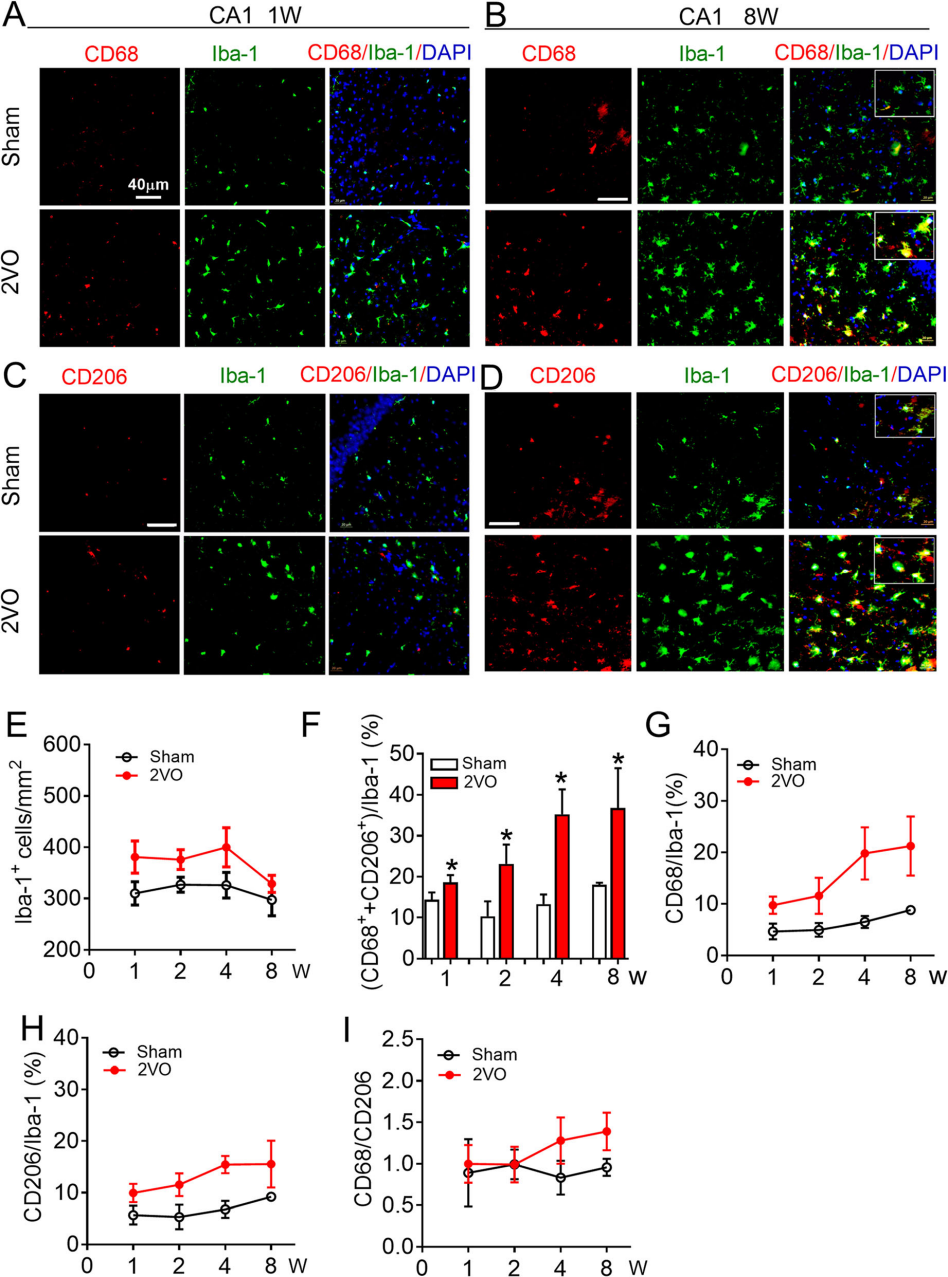
Microglial/macrophage activation and polarization in the CA1 region of 2VO rats. **a**, **b** Representative images of CD68 expression in Iba-1^+^ cells in hippocampal CA1 region 2VO rats at 1 week (**a**) and 8 weeks (**b**) by immunofluorescence staining. **c**, **d** Representative images of CD206 expression in Iba-1^+^cells in hippocampal CA1 region 2VO rats at 1 week (**c**) and 8 weeks (**d**) by immunofluorescence staining. Scale: 40 μm. **e** Quantification of the Iba-1^+^ cell number in hippocampal CA1 region. **f** Quantification of the total polarized microglia/macrophage number in hippocampal CA1 region. **g** Quantification of the ratio of CD68 in Iba-1^+^ cells in hippocampal CA1 region. **h** Quantification of the ratio of CD206 in Iba-1^+^ cells in hippocampal CA1 region. **i** Quantification of the ratio of CD68 and CD206 in hippocampal CA1 region. Values are mean ± SD, *n* = 9 slices from 3 animals per group, *P < 0.05 vs sham group, Student’s *t* test

In order to investigate how CBH affects the activation of hippocampal microglia/macrophage, we generated a rat model of CBH through 2VO surgery. First, we evaluated the polarization of microglia/macrophage in the hippocampus in rats at 1, 2, 4, and 8 weeks after 2VO surgery. As illustrated in Fig. 1a–e, immunofluorescence staining showed that the number of microglial/macrophage marker Iba-1 (Iba-1^+^ cells)-positive cells in the dentate gyrus (DG) region was significantly increased at 1 week and gradually decreased to the same level as the sham group at 8 weeks. Since activated microglia/macrophage can be polarized to the cytotoxic M1 phenotype and the pro-repair M2 phenotype [34], we next compared activated microglia/macrophage between two groups by analyzing the ratio of polarized microglia/macrophage relative to total Iba-1^+^ cells. Using immunofluorescence staining, the number of the co-localization of the M1-associated marker CD68 or the M2-associated marker CD206 with Iba-1 were counted. The data showed that the total percentage of polarized microglia/macrophage gradually increased from 2 weeks (30.63 ± 3.46%) to 8 weeks (52.42 ± 8.59%) of 2VO rats; however, they were unchanged in sham group (Fig. 1f). To clarify the characteristic phenotype of the activated microglia/macrophage, the percentages of CD68-positive (CD68^+^) and CD206-positive (CD206^+^) microglia/macrophage were analyzed, respectively. Compared with the sham group, the percentage of both phenotypes of microglia/macrophage gradually increased in the DG region of CBH rats from 2 weeks to 8 weeks after surgery. As shown in Fig. 1g, h, the percentage of CD68^+^ and CD206^+^microglia/macrophage were the same between sham and 2VO rats at 1 week after surgery (for CD68^+^: 10.15 ± 3.61% in sham, 9.71 ± 1.69%% in 2VO; for CD206^+^: 10.11 ± 1.69% in sham, 10.9 ± 1.92% in 2VO). However, they were significantly higher in 2VO rats at 8 weeks than they were in age-matched sham rats (for CD68^+^: 11.73 ± 6.25% in sham, 33.14 ± 5.27% in 2VO; for CD206^+^: 12.34 ± 2.15% in sham, 19.27 ± 1.45% in 2VO). Subsequent, the ratio of CD68/CD206 was statistic to better understand the dynamic changes in microglial/macrophage polarization. The ratio of CD68/CD206 higher than 1 indicates that microglial/macrophage polarization towards to the M1 phenotype. The data showed that the ratio of CD68/CD206 was the same between 2VO and sham rats at 1 week and 2 week. The ratio began to increase in 2VO rats from 4 weeks after surgery and reached the highest value at 8 weeks, which was 1.76 ± 0.30-fold greater than that in the sham rats at 8 weeks (0.92 ± 0.25) (Fig. 1i). Similar to what was observed in the DG, the number of Iba-1^+^ cells in CA1 region of 2VO rats also increased significantly at 1 week and gradually decreased to the same level as sham group at 8 weeks (Fig. 2a–e). Different from that observed in the DG region, the total number of polarized microglia/macrophage in CA1 region increased gradually from 1 week to 8 weeks (Fig. 2f). Specificity, the percentage of total CD68^+^ and CD206^+^ microglia/macrophage was higher in 2VO rats than age-matched sham rats at 1 week after surgery (14.35 ± 1.80% in sham vs. 18.61 ± 1.78% in 2VO, *P* = 0.043) and this phenomenon lasted for 8 weeks (18.03 ± 0.49% in sham, 36.79 ± 9.67% in 2VO, *P* = 0.0168). Interestingly, although the number of CD68^+^ microglia/macrophage was also higher than the number of CD206^+^ microglia/macrophage in the CA1 region of 2VO rats at 8 weeks (Fig. 2g, h), the ratio of CD68/CD206 in the CA1 region (Fig. 2i, 1.38 ± 0.23-fold) was lower than that in the DG region (Fig. 1i, 1.76 ± 0.3-fold ); this suggests that CBH-induced microglial/macrophage polarization is more sensitive in the DG region than CA1. Taken together, these results suggested that the activation of hippocampal microglia/macrophages runs through the whole process of CBH from an early stage (1 week) to 8 weeks. However, polarization transitioned to a more detrimental M1 phenotype at 8 weeks after being balanced between the M1 and M2 phenotypes at 1 week and 2 weeks after 2VO surgery.

**Fig. 3:**
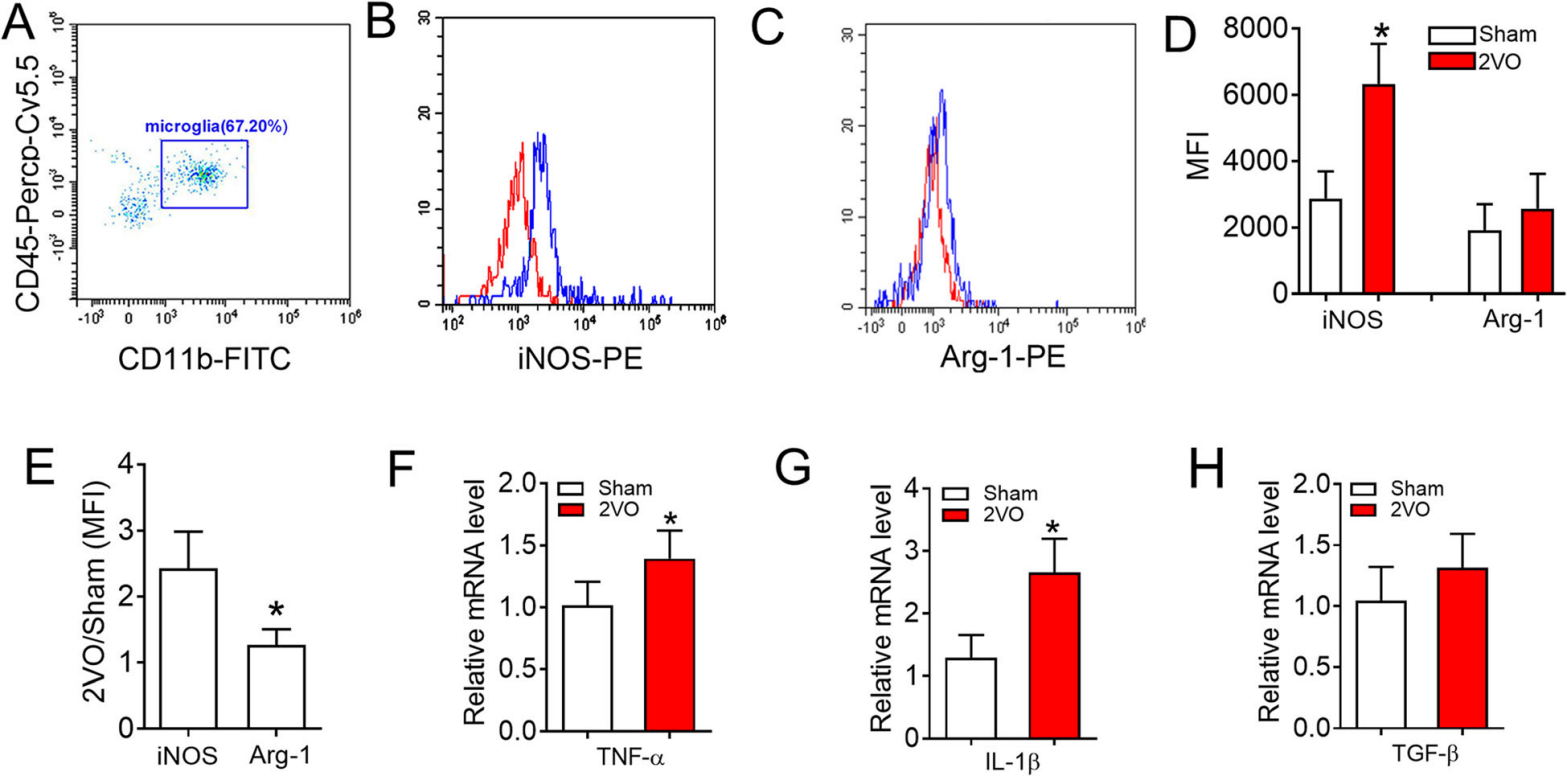
CBH induces microglial polarization toward M1 phenotype and inflammatory responses in 2VO rats after 8 weeks. **a** Representative dot plots of CD11b/CD45 cells detected by flow cytometry. Microglia cells within the gate were used for iNOS/Arg-1 analysis. **b** Representative histograms of the mean fluorescent intensity (MFI) for iNOS in sham (red) and 2VO (blue) rats. **c** Representative histograms of the MFI for Arg-1 in sham and 2VO rats. **d** Quantification of MFI for iNOS/Arg-1 from sham and 2VO rats. Bars represent the mean ± SD. *n* = 3, cells were pooled from 3 rats per group for a total of 3 separate experiments for each time point. *P < 0.05 vs sham group, Student’s *t* test. **e** The MFI ratio between 2VO and sham rats for the expression of iNOS/Arg-1. Bars represent the mean ± SD. *n* = 3, cells were pooled from 3 rats per group for a total of 3 separate experiments for each time point. *P < 0.05 vs iNOS, Student’s *t* test. **f** The mRNA expression of TNF-α in the hippocampus of 2VO rats. **g** The mRNA expression of IL-1β in the hippocampus of 2VO rats. **h** The mRNA expression of TGF-β in the hippocampus of 2VO rats. Bars represent the mean ± SD. *n* = 6. *P < 0.05 vs sham group, Student’s *t* test

To further verify that microglial polarization tended to be associated with the M1 phenotype at 8 weeks after 2VO surgery, flow cytometry analysis was performed using a variety of microglial markers to assess the status of the M1 and M2 phenotypes. First, we sorted microglia using CD11b^+^/CD45^low^ as a marker (Fig. 3a and Supplementary Fig. S1) [35, 36]. Then, the mean fluorescence intensities (MFI) of iNOS (M1 marker) and Arginase-1 (Arg-1, M2 marker) were detected [11, 13, 37]. The data revealed a significantly increased percentage of iNOS expression in microglial cells of 2VO rats than in microglial cells of sham rats (Fig. 3b, d, 2878.87 ± 819.84 vs. 6327.33 ± 1216.83, *P* = 0.0152). However, the level of Arg-1 was unchanged (Fig. 3c, d, 1913.4 ± 788.17 vs. 2573.13 ± 1049.63, *P* = 0.4331). This phenomenon was further verified by the higher ratio of iNOS between 2VO and sham groups than Arg-1 (Fig. 3e). Currently, proinflammatory cytokines (TNF-α and IL-1β) are thought to be produced by M1 microglia/macrophage while M2 microglia/macrophage can secrete anti-inflammatory cytokines and trophic factors, such as TGF-β [38, 39]. QRT-PCR analysis showed that the mRNA levels of the proinflammatory cytokines TNF-α and IL-1β were elevated in the hippocampus of 2VO rats compared with sham rats (Fig. 3f, g). However, the mRNA level of the anti-inflammatory cytokine TGF-β was not increased (Fig. 3h). All these data suggest that the polarization of hippocampal microglial in 2VO is skewed towards the M1-type phenotype at week 8.

### Knockdown of *miR-195* polarizes microglia/macrophage towards the detrimental M1 phenotype

**Fig. 4:**
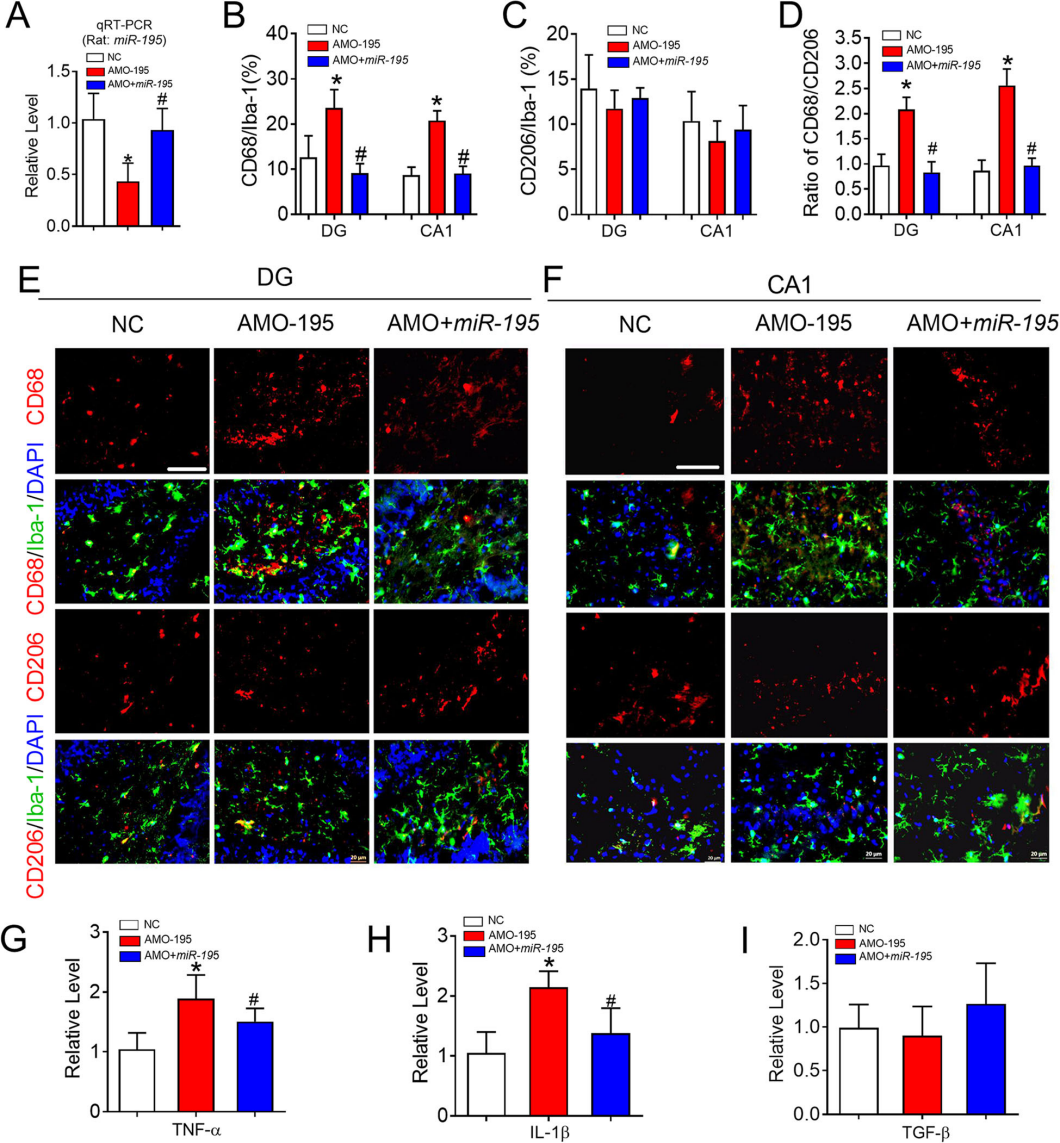
Knockdown of *miR-195* prime microglial/macrophage polarization to M1 phenotype in rats. **a** *MiR-195* expression was detected by qRT-PCR in the hippocampus of rats following the stereotaxic injection of lenti-pre-AMO-*miR-195* and/or lenti-pre-m*iR-195* into CA1 region. Bars represent the mean ± SD. *n* = 6. *P < 0.05 vs NC group, ^#^*P* < 0.05 vs AMO-195. **b** Changes of *miR-195* affect the percentage of CD68 in the Iba-1^+^cells in hippocampal DG, CA1 region of rats. **c** Changes of *miR-195* has no effects on the percentage of CD206 in the Iba-1^+^cells in hippocampal DG, CA1 region of rats. **d** Changes of *miR-195* regulate the ratio of CD68/CD206 in the hippocampal DG and CA1 regions. Bars represent the mean ± SD. *n* = 9 slices from 3 animals per group, *P < 0.05 vs NC group, ^#^*P* < 0.05 vs AMO-195. **e**, **f** Representative images of CD68 and CD206 expression in the Iba-1^+^ cells of rat hippocampal DG (**e**) and CA1 (**f**) region following the stereotaxic injection of lenti-pre-AMO-*miR-195* and/or lenti-pre-*miR-195* into CA1 region by immunofluorescence staining. The scale bar was 40 μm. **g** The mRNA expression of TNF-α in the hippocampus of rats following the stereotaxic injection of lenti-pre-AMO-*miR-195* and/or lenti-pre-*miR-195* into CA1 region. **h** The mRNA expression of IL-1β in the hippocampus of rats following the stereotaxic injection of lenti-pre-AMO-*miR-195* and/or lenti-pre-*miR-195* into CA1 region. **i** The mRNA expression of TGF-β in the hippocampus of rats following the stereotaxic injection of lenti-pre-AMO-*miR-195* and/or lenti-pre-*miR-195* into CA1 region. Bars represent the mean ± SD. *n* = 6. *P < 0.05 vs NC group, ^#^*P* < 0.05 vs AMO-195. All data were analyzed using one-way ANOVA followed by Tukey test

Previous studies have reported that microRNA-195 (*miR-195*) can reduce polarization of M1-like macrophage and inhibit the inflammatory pathway in the peripheral nervous system [28, 29]. In addition, our previous study found that *miR-195* expression was downregulated in the hippocampus of CBH rats [5, 7]. Therefore, we speculated that *miR-195* might have the potential to polarize microglia/macrophage in the hippocampus. To test this hypothesis, *miR-195* oligonucleotide fragments (lenti-pre-*miR-195*) and an anti-*miR-195* oligonucleotide fragments (lenti-pre-AMO-*miR-195*) packaged in a lentivirus vector were delivered directly into the bilateral hippocampal CA1 region of rats to examine the role of *miR-195* in microglial/macrophage polarization. The successful delivery of lenti-pre-AMO-*miR-195* and lenti-pre-*miR-195* was verified by the detection of *miR-195* levels by qRT-PCR (Fig. 4a, *F* = 11.50, *P* = 0.003). Immunofluorescence analysis showed that lenti-pre-AMO-*miR-195* application resulted in an increased number of CD68^+^ microglia/macrophage cells in both the DG and CA1 region compared with that in rats injected with negative control (NC) and that this effect was prevented by co-injection of lenti-pre-*miR-195* (Fig. 4b, e, and f, DG: *F* = 123.5, *P* = 0.0031; CA1: *F* = 28.57, *P* = 0.017). Interestingly, the injection of lenti-pre-AMO-*miR-195* did not affect the number of CD206^+^ microglia/macrophage in the DG and CA1 regions (Fig. 4c, e, and f, DG: *F* = 0.4743, *P* = 0.6265; CA1: *F* = 1.689, *P* = 0.3127). Accordingly, the ratio of CD68/CD206 was markedly increased after lenti-pre-AMO-*miR-195* was injected into the hippocampus, and this effect was reversed by co-injection of lenti-pre-*miR-195* (Fig. 4d, DG: *F* = 21.76, *P* = 0.0394; CA1: *F* = 34.33, *P* = 0.0179). In addition, we observed that knockdown of *miR-195 *induced significant increases in the levels of TNF-α and IL-1β, which were inhibited by *miR-195* gain of function (Fig. 4g, *F* = 10.26, *P* = 0.0103 and *H*, *F* = 20.59, *P* = 0.0030). However, downregulation of *miR-195* had no effect on TGF-β and this effect was not influenced by the upregulation of *miR-195* (Fig. 4i, *F* = 1.044, *P* = 0.3824). These data suggested that knockdown of *miR-195* can drive microglial/macrophage polarization towards the M1 phenotype in the rat hippocampus.

To further confirm this observation, we transfected *miR-195* mimics and the *miR-195* antisense oligonucleotide AMO-195 directly into BV2 microglial cells. As illustrated in Supplementary Fig. S2A & B, transfection with *miR-195* mimics did not affect the percentage of CD68^+^ microglia cells, but the antisense oligonucleotide AMO-195 induced a marked increase in the number of CD68^+^ microglia cells, which was prevented by co-transfection with *miR-195* mimics. However, transfection with the *miR-195* mimics or AMO-195 and co-transfection with the *miR-195* mimics and AMO-195 did not affect the number of CD206^+^ microglial cells (Supplementary Fig. S2A & C). Furthermore, it has been reported that microglia can be primed towards the M1 phenotype by lipopolysaccharide (LPS) [39]. Thus, we added LPS (100 μg mL^−1^) to the cultured BV2 cells for 24 h and observed that the number of CD68^+^ microglial cells but not that of CD206^+^ microglial cells was increased (Supplementary Fig. S2B & C); additionally, this effect was prevented by co-administration of the *miR-195* mimics. By analyzing the ratio of CD68/CD206, we found that AMO-195 administration had the same effect as LPS on M1 microglial polarization and that the effects of both these agents were blocked by co-transfection with the *miR-195* mimics (Supplementary Fig. S2D).

### CX3CL1-CX3CR1 signalling involving *miR-195* regulated microglial/macrophage polarization

**Fig. 5:**
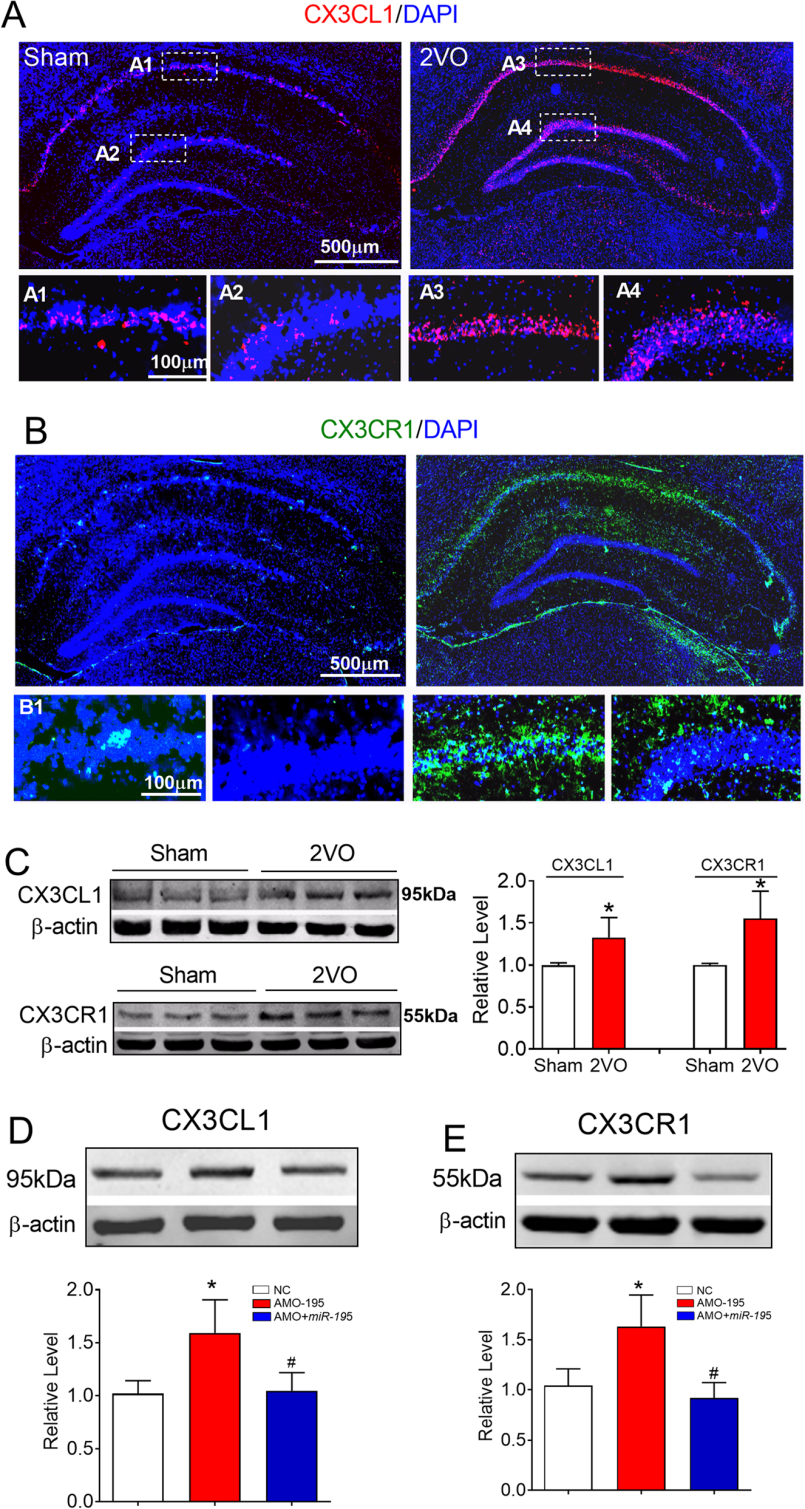
Upregulation of the expression of CX3CL1 and CX3CR1 in the hippocampus of 2VO and *miR-195* loss-of-function rats. **a**, **b** Expression of CX3CL1 (red) and CX3CR1 (green) in the hippocampus of sham and 2VO rats was shown by immunofluorescence staining. The scale bar is 500 um. **c** Expression of CX3CL1 and CX3CR1 in the hippocampus of sham and 2VO rats detected by western blot technique. Bars represent the mean ± SD, *n* = 6, *P < 0.05 vs sham group, Student’s *t* test. **d** Lenti-pre-AMO-*miR-195* upregulated the expression of CX3CL1 in rat hippocampus. Mean ± SD, *n* = 6, *P < 0.05 vs sham; ^#^*P* < 0.05 vs lenti-pre-AMO-miR-195. Data were analyzed using one-way ANOVA followed by Tukey test. **e** Lenti-pre-AMO-*miR-195* upregulated the expression of CX3CR1 in the rat hippocampus. Mean ± SD, *n* = 6, *P < 0.05 vs sham; ^#^*P* < 0.05 vs lenti-pre-AMO-*miR-195*. Data were analyzed using one-way ANOVA followed by Tukey test

CX3CL1-CX3CR1 signaling plays an important role in neuroinflammatory diseases of the CNS [40]. A previous study demonstrated that the expression of CX3CL1 and CX3CR1 is increased in rats early after ischemic stroke and that reducing the expression of CX3CL1 and CX3CR1 is beneficial for the recovery of neurological function [41]. Importantly, it has been found that CX3CL1/CX3CR1-mediated microglial activation can promote the generation of TNF-α and IL-1β, inducing a detrimental effect in the brains of ischaemic mice in the early stage [42]. Since M1 microglia typically release destructive proinflammatory mediators such as TNF-α and IL-1β [38], the levels of TNF-α and IL-1β expression were significantly increased in 2VO rats compared with sham rats (Fig. 3). We hence hypothesized that CX3CL1-CX3CR1 signaling might be involved in microglial polarization in CBH rats. As predicted, the immunofluorescence signals of CX3CL1 and CX3CR1 in the hippocampus of 2VO rats at 8 weeks were much higher than those in sham rats (Fig. 5a, b). Western blotting analysis also verified the increase in the protein expression of CX3CL1 and CX3CR1 in the hippocampus of 2VO rats compared with sham rats (Fig. 5c).

It has been reported that *miR-195* protects against focal acute ischaemic stroke by targeting CX3CR1 but not CX3CL1 in mice, although both CX3CL1 and CX3CR1 are direct targets of *miR-195* [27]. However, the effect of *miR-195* on microglial/macrophage polarization in the rat hippocampus following chronic mild brain ischaemia is unknown. We first evaluated the effect of *miR-195* on CX3CR1 and CX3CL1 expression. We found that knockdown of *miR-195* by injection of lenti-pre-AMO-*miR-195* induced a significant increase in CX3CL1 (Fig. 5D, *F* = 14.74, *P* = 0.0088) and CX3CR1 (Fig. 5e, *F* = 19.91, *P* = 0.0029) expression and that this change was inhibited by co-injection of lenti-pre-*miR-195*.

**Fig. 6:**
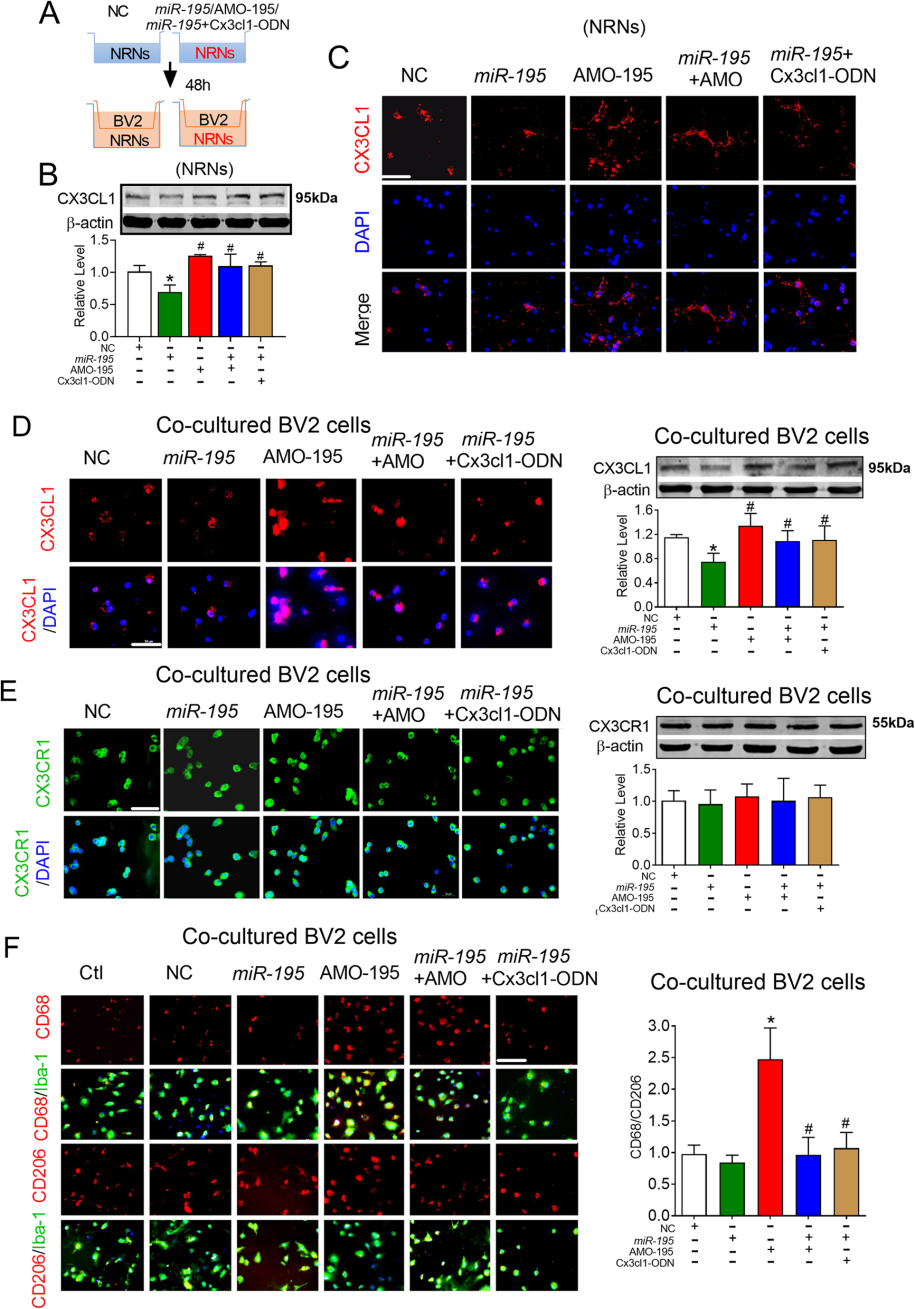
Knockdown of *miR-195* induced microglial polarized toward M1 phenotype-dependent on the CX3CL1-CX3CR1 signaling pathway in vitro. **a** Schematic diagram of the neuron-microglia cell co-culture system. Neurons were transfected with *miR-195*, AMO-195, *miR-195* + AMO-195, Cx3cl1-ODN, or NC for 48 h. Then, BV2 cells were seeded in the top compartment of the transwell with the NRNs were cultured in the bottom compartment. Subsequently, BV2 cells were co-cultured with NRNs for 24 h. **b**, **c** The effects of *miR-195* on endogenous CX3CL1 expression in NRNs by western blotting (**b**) and immunofluorescence staining (**c**) after the NRNs were transfected with *miR-195*, AMO-195, *miR-195* + AMO-195,* miR-195*+ Cx3cl1-ODN, or NC. **d** *MiR-195* downregulated CX3CL1 expression in co-cultured BV2 cells assessed by immunofluorescence staining and western blotting. **e** *MiR-195* did not affect CX3CR1 expression in co-cultured BV2 cells evaluated assessed by immunofluorescence staining and western blotting. **f** Downregulating *miR-195* increased the ratio of CD68/CD206 in Iba-1+ cells in co-cultured BV2 cells. Bars represent the mean ± SD, *n* = 3 batches of cell culture. *P < 0.05 vs NC; ^#^*P* < 0.05 vs AMO-195. Scale bar: 40 μm. All data were analyzed using one-way ANOVA followed by Tukey test

The CX3CL1-CX3CR1 pathway is a critical signaling pathway for cellular communication between neurons and microglia [1, 43]. To further clarify whether the CX3CL1-CX3CR1 signaling pathway participates in *miR-195* knockdown-induced microglial polarization, we established a neuronal-microglial co-cultured model. In this co-culture system (Fig. 6a), we first transfected primary-cultured NRNs with *miR-195* mimics and AMO-195 for 48 h and co-cultured microglia with NRNs for 24 h. The western blot results showed that AMO-195 upregulated the expression of CX3CL1 on NRNs and that this effect was prevented by co-transfection with the *miR-195* mimics (Fig. 6b, *F* = 11.874, *P* = 0.0369). To further clarify the direct effect of *miR-195* on CX3CL1 expression, we designed a miRNA-masking antisense oligodeoxynucleotides (ODN) of the *Cx3cl1* gene to mask the *miR-195* binding site of the *Cx3cl1* gene. The data showed that co-transfection of NRNs with Cx3cl1-ODN (240-246 bp region of the 3′UTR) and *miR-195* blocked the inhibitory effects of *miR-195* on CX3CL1 expression (Fig. 6b). The results were consistent with immunofluorescence analysis (Fig. 6c). Communication between neurons and microglia cells is through the binding of the chemotactic factor CX3CL1 released by neurons to CX3CR1 on microglial cells, which elicit the activation of microglia [44, 45]. Therefore, we monitored the expression of CX3CL1 in co-cultured BV2 microglial cells. We found that the *miR-195* mimics inhibited the expression of CX3CL1 while AMO-195 increased CX3CL1 expression and that these changes did not occur when NRNs were co-transfected with the *miR-195* mimics and AMO-195 or Cx3cl1-ODN (Fig. 6d, *F* = 9.953, *P* = 0.0465). However, the expression of CX3CR1 on co-cultured BV2 cells was not changed in these groups (Fig. 6e, *F* = 0.2971, *P* = 0.7429). Importantly, by analyzing the microglial polarization of co-cultured BV2 cells, we found that AMO-195 transfection induced a marked increase in the CD68/CD206 ratio and that this effect was prevented by the *miR-195* mimics and Cx3cl1-ODN (Fig. 6f, *F* = 22.12, *P* = 0.0101). This phenomenon suggests that blocking the release of CX3CL1 from NRNs can prevent the M1 polarization of microglia.

**Fig. 7:**
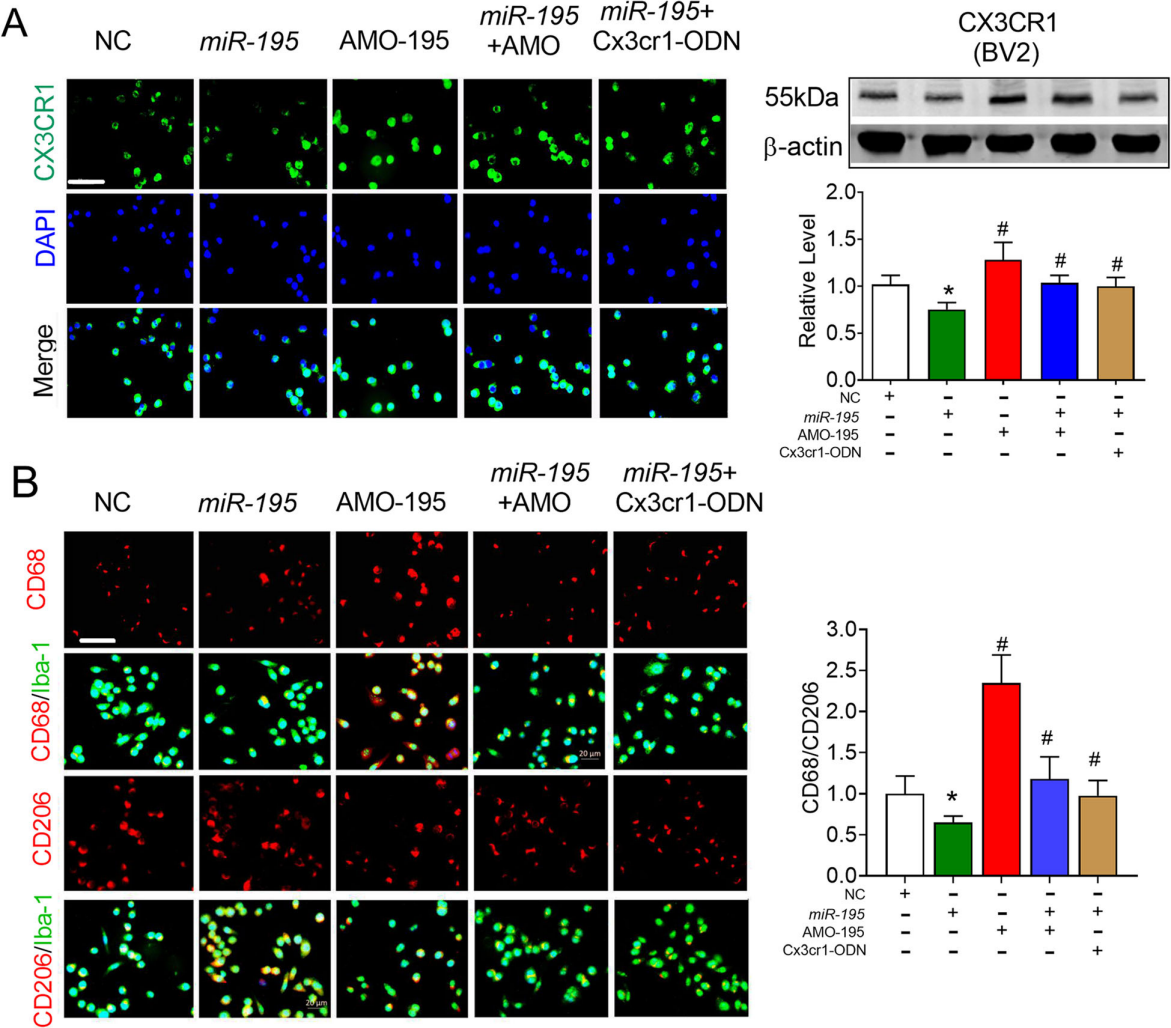
Knockdown of *miR-195* directly in BV2 cells induced microglial polarized toward M1 phenotype dependent on the CX3CR1 expression. **a** The effects of miR-195 on endogenous CX3CR1 expression in BV2 cells by immunofluorescence staining and western blotting after the BV2 cells were transfected with miR-195, AMO-195, *miR-195* + AMO-195, *miR-195* + Cx3cr1-ODN, or NC. **b** Downregulating *miR-195* directly in the BV2 cells increased the ratio of CD68/CD206 in Iba-1+ cells. Bars represent the mean ± SD, *n* = 3 batches of cell culture. *P < 0.05 vs NC; ^#^*P* < 0.05 vs *miR-195*. Scale bar: 40 μm. All data were analyzed using one-way ANOVA followed by Tukey test

Next, we directly transfected BV2 microglial cells with *miR-195* and AMO-195 to observe the regulatory effect of *miR-195* on microglial polarization and the levels of CX3CR1, which has been reported to be expressed on microglial cells [44]. Similar to the effect of *miR-195* on CX3CL1 expressed on NRNs, *miR-195* inhibited CX3CR1 expression on BV2 microglial cells, and this effect was reversed by AMO-195 and Cx3cr1-ODN (Fig. 7a, *F* = 16.09, *P* = 0.0012); this suggests that *miR-195* regulates CX3CR1 expression by targeting the 3′UTR (the 1236-1242 bp region of the 3′UTR) of the *Cx3cr1* gene. Furthermore, we observed transfecting AMO-195 into BV2 cells induced the M1 microglial polarization, which was prevented by the *miR-195* mimics and Cx3cr1-ODN (Fig. 7b, *F* = 30.99, *P* = 0.0292).

### *MiR-195* prevents microglial/macrophage polarization towards the M1 phenotype induced by CBH

**Fig. 8:**
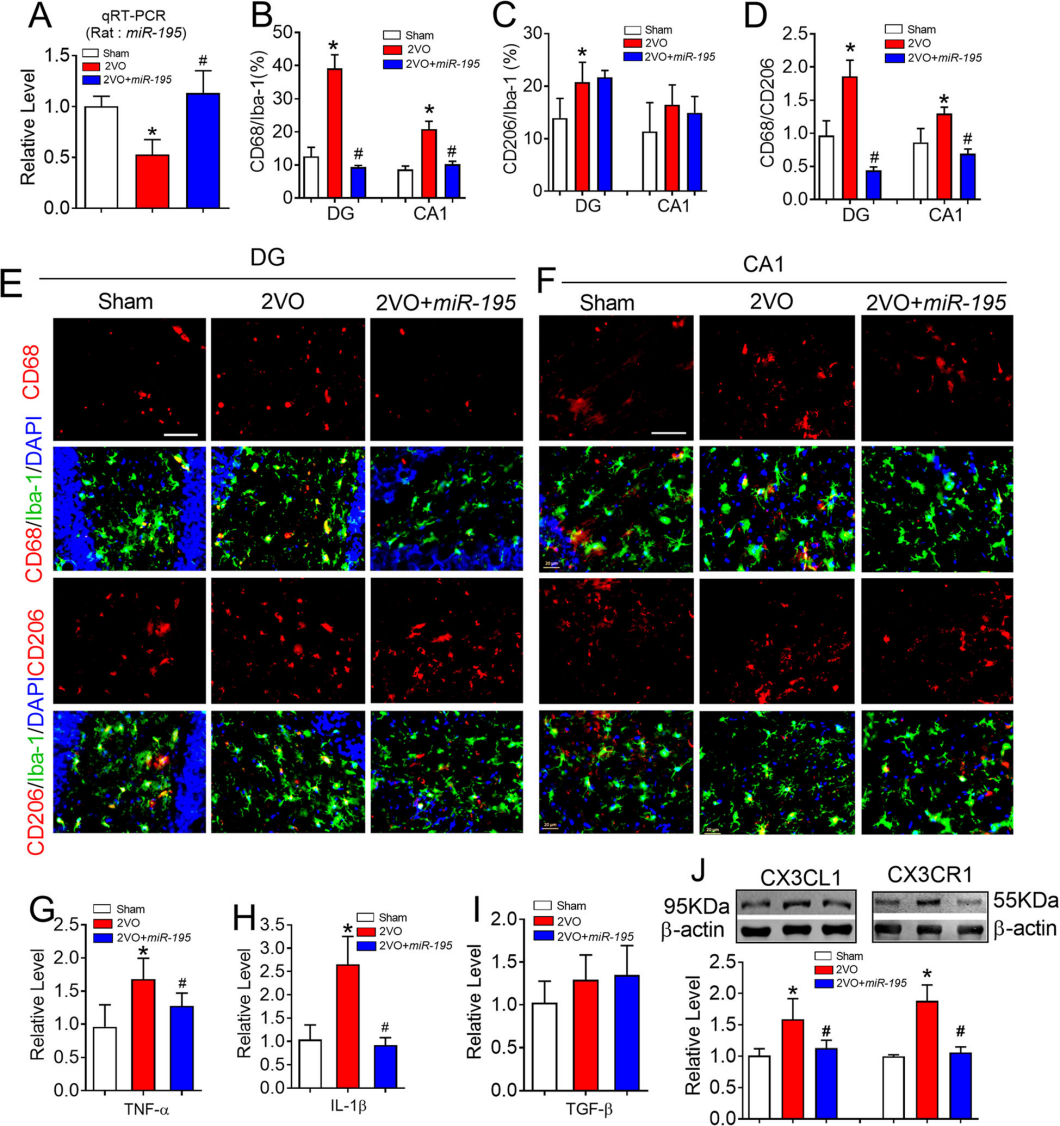
*MiR-195* prevented the microglial/macrophage polarization to M1 phenotype induced by 2VO surgery. **a** *MiR-195* expression in the hippocampus of 2VO rats at 8 weeks with or without lenti-pre-*miR-195 *treatment was detected by qRT-PCR. Bars represent the mean ± SD. *n* = 6. *P < 0.05 vs sham group, ^#^*P* < 0.05 vs 2VO. **b** *MiR-195* decreased the percentage of CD68 in the Iba-1^+^ cells in hippocampal DG and CA1 region of 2VO rats. **c** *MiR-195* did not affect the percentage of CD206 in the Iba-1^+^ cells either in hippocampal DG region or CA1 region of 2VO rats. **d** *MiR-195* reversed the increased ratio of CD68/CD206 in the hippocampal DG and CA1 regions of 2VO rats. Bars represent the mean ± SD, *n* = 9 slices from 3 animals per group. *P < 0.05 vs sham group, ^#^*P* < 0.05 vs 2VO. **e**, **f** Representative images of CD68 and CD206 expression in the Iba-1^+^ cells of hippocampal DG (**e**) and CA1 (**f**) region of 2VO rats following the stereotaxic injection of lenti-pre-miR-195 into CA1 region. The scale bar was 40 μm. **g** Lenti-pre-*miR-195* injection reversed the increased mRNA level of TNF-α in the hippocampus of 2VO rats. **h** Lenti-pre-*miR-195* injection reversed the increased mRNA level of IL-1β in the hippocampus of 2VO rats. **i** Lenti-pre-*miR-195* injection did not affect TGF-β level in the hippocampus of 2VO rats. **j** Lenti-pre-*miR-195* injection inhibited the increased CX3CL1 and CX3CR1 in the hippocampus of 2VO rats. Bars represent the mean ± SD, *n* = 6. *P < 0.05 vs sham group, ^#^*P* < 0.05 vs 2VO. All data were analyzed using one-way ANOVA followed by Tukey test

We next assessed whether *miR-195* indeed plays a beneficial role in 2VO-induced microglial/macrophage polarization. To address this issue, lenti-pre-*miR-195* was injected into the hippocampal CA1 region of 2VO rats. Using real-time PCR analysis, we found that lenti-pre-*miR-195* injection increased the level of *miR-195* in the hippocampus of 2VO rats, suggesting a successful delivery of *miR-195* in 2VO rats (Fig. 8a, *F* = 20.86, *P* = 0.0017). As predicted, injection of lenti-pre-*miR-195* into the hippocampus of 2VO rats effectively reversed 2VO-induced a high number of CD68^+^ microglia/macrophage (Fig. 8b, e, and f, DG: *F* = 47.12, *P* = 0.0179; CA1: *F* = 21.74, *P* = 0.0430) and an increase in the ratio of CD68/CD206 in both the DG and CA1 region (Fig. 8d, DG: *F* = 58.46, *P* = 0.0111; CA1: *F* = 11.90, *P* = 0.0490), but did not significantly affect the number of CD206^+^ microglia/macrophage (Fig. 8c, e, and f, DG: *F* = 5.372, *P* = 0.0818; CA1: *F* = 1.078, *P* = 0.4109). Consistent with these results*,* injection of lenti-pre-*miR-195* prevented an increase in the mRNA levels of TNF-α (Fig. 8g, *F* = 6.067, *P* = 0.0256) and IL-1β (Fig. 8h, *F* = 25.32, *P* = 0.0031) but did not affect the mRNA level of TGF-β (Fig. 8i, *F* = 1.940, *P* = 0.2147). Accordingly, lenti-pre-*miR-195* significantly inhibited the elevation of CX3CL1 (*F* = 22.55, *P* = 0.0035) and CX3CR1 (*F* = 79.22, *P* < 0.0001) expression in the hippocampus of 2VO rats (Fig. 8j).

## Discussion

Microglia play an important role in neurodegenerative diseases [1, 2]. Meanwhile, CBH has been found to be a preclinical phase of AD and VaD [3, 4]. However, how CBH influences the neuroimmune process is unknown. Here, we first reported that CBH initiates microglial/macrophage activation in the rat hippocampus from 1 week to 8 weeks after 2VO surgery. The balance of microglial/macrophage polarization towards the M1 and M2 phenotypes was shifted towards the M1 phenotype at 8 weeks following CBH. Further study demonstrated that CBH downregulated the expression of *miR-195* and this downregulated expression of *miR-195* posttranscriptional upregulated the level of CX3CL1/CX3CR1, thereby promoting a pro-inflammatory microglial phenotype. This study provides evidence that *miR-195* treatment may be a good strategy for preventing CBH-induced detrimental M1 phenotype.

It has been found that activated microglia and/or macrophages represent spectrum phenotypes after injury [13–15]. For example, activated M2 phenotype has three subtypes including M2a, M2b, and M2c and perform distinct biological functions [46]. However, since their function in CNS injuries have not yet been characterized, the broadly classified M1 and M2 phenotype remains useful for understanding the function of microglia/macrophage in various brain diseases [15]. Previous studies have reported that microglia/macrophages response to stroke and TBI is dynamic, exhibiting an early M2 phenotype, followed by a transition to M1 phenotype [14, 20], which suggesting that manipulating the polarization of microglia/macrophage might be a promising therapeutic strategy for brain repair. However, studies on the process of microglial/macrophage polarization during chronic cerebral ischemia are limited. In the current study, we used three methods to evaluate how CBH affects the microglial/macrophage polarization process. First, immunofluorescence technique was performed by using Iba-1, CD68, and CD206 as the biomarkers. We found that, like acute and severe brain injury, mild chronic brain ischemia induced by CBH also elicited dynamic microglial/macrophage responses. However, in contrast to what has been observed in severe brain injury, we found that the ratio of CD68/CD206 in Iba-1^+^ cells in the hippocampus of 2VO rats was approximately 1.0 at 1 week and 2 weeks after surgery but gradually increased by 4 weeks and 8 weeks. The results demonstrated that there was a balance between microglial/macrophage polarization towards the M1 and M2 phenotypes in the early stage of mild brain ischemia followed by a switch towards the detrimental M1 phenotype. Since Iba-1 (a calcium-binding groups of protein), CD68 (a phagocytic marker), and CD206 (a mannitol receptor) are not specific microglia markers and they are also expressed by infiltrating macrophages and monocytes [47], we then used flow cytometry to sort microglia using CD11b^+^/ CD45^low^ as a marker and detected increased iNOS level and unchanged Arg-1 level. This phenomenon was further proven by significant increase in the levels of TNF-α and IL-1β and unchanged TGF-β at 8 weeks. All these results suggested that increased M1 phenotype in the hippocampus of CBH rats at 8 weeks.

Our previous studies revealed that CBH lasting for 8 weeks results in multiple AD-like phenotypes, including Aβ aggregation [5], tau hyperphosphorylation [6], inactivation of protein phosphatase-2A (PP2A) [48], and cell death [7] in rats. Interestingly, all these pathological changes can be regulated by a single microRNA, *miR-195*. It has been reported that *miR-195* targets the inflammatory protein IL-1β in macrophages [49]. Additionally, it reduces M1-like macrophage polarization [29]. In the present study, using the BV2 microglial cell line, we found that blocking endogenous *miR-195* by transfection with the antisense oligonucleotide AMO-195, which was similar to LPS, resulted in M1 phenotype microglial polarization. Transfection of the *miR-195* mimics inhibited the increase in the percentage of CD68^+^ cells that were induced by both AMO-195 and LPS administration. Furthermore, we demonstrated that knockdown of *miR-195* by injection of lenti-pre-AMO-*miR-195* into the hippocampus elicited a marked increase in CD68 expression in Iba-1^+^ cells but had no effect on CD206 expression, suggesting that *miR-195* loss of function can prime detrimental M1 microglial/macrophage polarization and that supplementation of *miR-195* by lenti-pre-*miR-195* injection into the hippocampus not only blocks the effects of lenti-pre-AMO-*miR-195* but also prevents 2VO-induced M1 microglial/macrophage polarization.

The CX3CL1/CX3CR1 signaling pathway plays a key role in the process of microglial polarization [21]. However, its function in ischemic brain injury is controversial [24, 25, 42, 50, 51]. Consistent with a previous study in ischemic stroke mice [27], we found that the expression of both CX3CL1 and CX3CR1 increased significantly in the hippocampus of rats and that this effect was mimicked by knockdown of *miR-195*. As we predicted, upregulation of *miR-195* by lenti-pre-*miR-195* injection directly into the CA1 region reversed the elevation of CX3CL1 and CX3CR1 expression in 2VO rats. CX3CL1 is expressed and secreted from neurons and binds to its receptor, CX3CR1, on the surface of microglia to further regulate microglial polarization [2, 40]. To clarify the role of the CX3CL1 and CX3CR1 proteins in *miR-195*-mediated M1 polarization in CBH rats, we established a neuronal-microglial co-cultured model. We found that transfecting NRNs with AMO-195 significantly upregulated the expression of CX3CL1 on NRNs without affecting CX3CR1 levels in co-cultured BV2 cells but significantly increased the percentage of Iba-1^+^ cells that were CD68^+^; these effects were prevented by co-transfection with *miR-195* mimics and Cx3cl1-ODN. This result suggest that CX3CL1 is the direct target of *miR-195* and mediates *miR-195*-mediated microglial polarization. To further evaluate the effect of CX3CR1 on *miR-195-*mediated microglial polarization, we delivered AMO-195 directly to BV2 microglia and observed that the expression of CX3CR1 on BV2 cells was upregulated and that there was an increased number of Iba-1^+^ cells that were CD68^+^; these effects were prevented by co-transfection with *miR-195* mimics and Cx3cr1-ODN. These data suggest that *miR-195* controls microglial polarization by governing CX3CL1-CX3CR1 signaling through the direct regulation of CX3CL1 and CX3CR1 expression.

## Conclusions

**Fig. 9.**
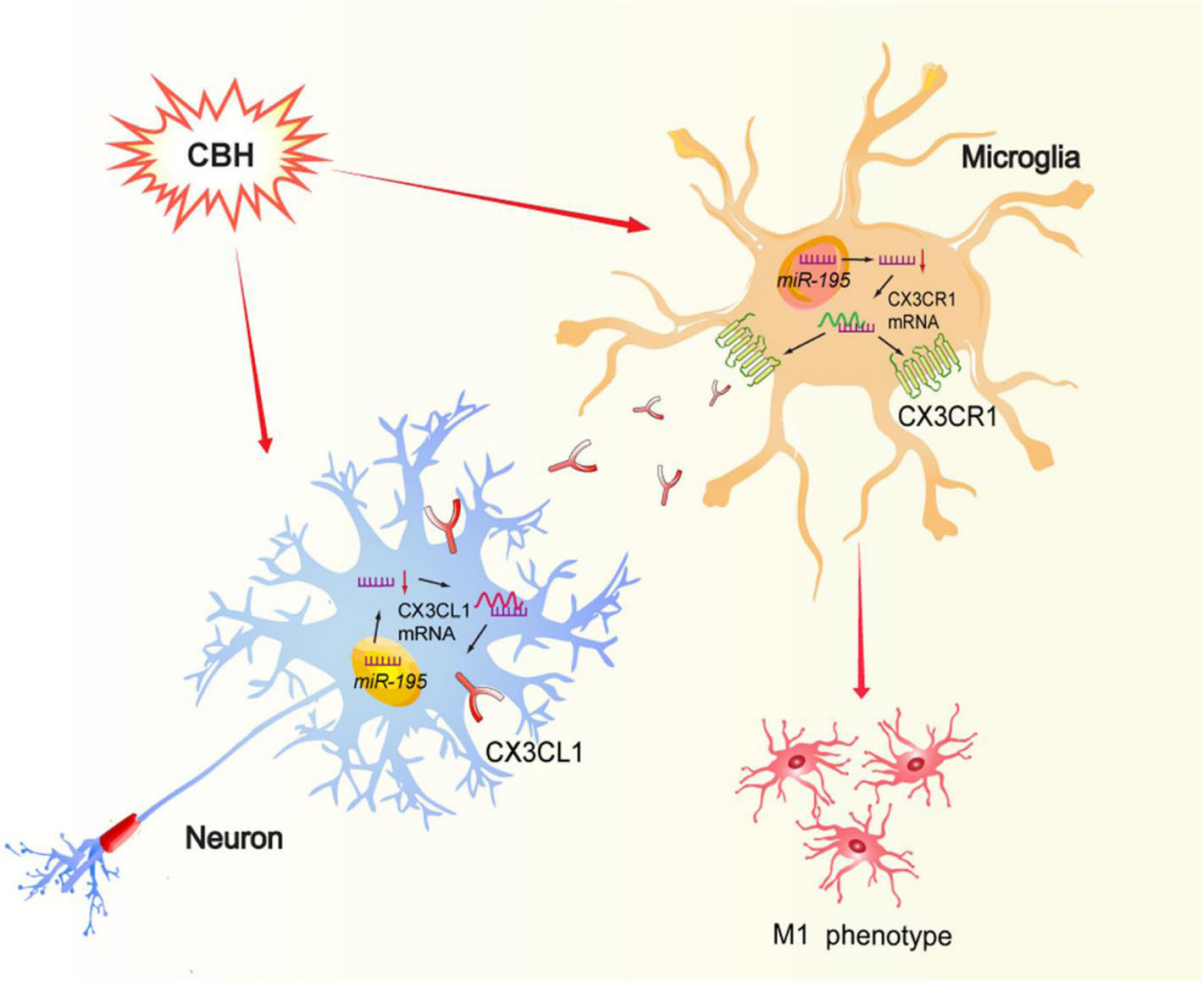
Schematic for the mechanisms of CBH induces M1 phenotype of microglial polarization. CBH downregulates *miR-195* and may induce microglial polarization toward to M1 phenotype in two ways: (1) downregulated *miR-195* upregulates the expression of CX3CL1 by binding with the 3′UTR of Cx3cl1 gene in neuron that were subsequently released and bound to its receptor CX3CR1 in microglia, which further results in the microglial polarization toward M1 phenotype; (2) downregulated *miR-195* upregulates the expression of CX3CR1 by binding with the 3′UTR of Cx3cr1 gene in microglia and results in the microglial polarization toward M1 phenotype directly

In summary, our study provides compelling evidence that downregulation of *miR-195* is involved in CBH-induced the polarization of hippocampal microglia/macrophage toward the M1 phenotype, which is mediated by activation of CX3CL1-CX3CR1 signaling between neurons and microglia (Fig. 9). The results suggest that increasing *miR-195* expression in the brain is a strategy for preventing the CBH-induced neuroimmune response and subsequent brain damage, such as cell death.

## Supplementary information

**Additional file 1: Table S1.** Animal Groups and Number of Rats Used in the Study.

**Additional file 2: Supplementary Fig. S1.** The gating strategy of microglia. Debris and aggregates were eliminated from the analysis by forward- and side-scatter characteristics (small plots). The alive myeloid cells were further identified by CD11b and CD45. Microglia were sorted using CD11b^+^/ CD45^low^ as a marker.

**Additional file 3: Supplementary Fig. S2.** *MiR-195* prevents LPS induced activation of microglia towards M1 profile of cultured BV2 cells. A. Representative images of CD68 or CD206 expression in Iba-1^+^BV2 cells by immunofluorescence staining after transfection of NC, *miR-195*, AMO-195, *miR-195*+AMO-*195*, LPS or LPS+*miR-195*.Scale bar: 40 μm. B&C. Quantification of the percentage of CD68 (B) or CD206 (C) in Iba-1^+^ BV2 cells. D. Quantification of the ratio of CD68/CD206 in BV2 cells. Bars represent the mean ± SD.; n= 9 from 3 batches of cell culture. *P<0.05 *vs* NC; ^#^*P*<0.05 *vs* AMO-195; ^$^*P*<0.05 *vs* LPS. All data were analyzed using one-way ANOVA followed by Tukey test.

## Data Availability

All data used during the current study available from the corresponding author on reasonable request.

## Abbreviations

AD: Alzheimer’s disease
Arg-1: Arginase-1
BSA: Bovine serum albumin
CBH: Chronic brain hypoperfusion
CCCI: Chronic cerebral circulation insufficiency
DG: Dentate gyrus
FBS: Fetal bovine serum
HCC: Hepatocellular carcinoma
HD: Huntingdon’s disease
ICH: Intracerebral hemorrhage
LPS: Lipopolysaccharide
*miR-195*: MicroRNA-*195*
MFI: Mean fluorescence intensity
NRNs: Neonatal rat neurons
ODN: Oligodeoxynucleotides
PD: Parkinson’s disease
PFA: Paraformaldehyde
PP2A: Protein phosphatase-2A
ROS: Reactive oxygen species
SD rats: Sprague Dawley rats
TBI: Traumatic brain injury
TNF-α: Tumor necrosis factor α
2VO: Bilateral common carotid artery occlusion

## References

[1] Heneka MT (2019). Microglia take centre stage in neurodegenerative disease. Nat Rev Immunol.

[2] Hickman SE, Izzy S, Sen P, Morsett L, El Khoury J (2018). Microglia in neurodegeneration. Nat Neurosci.

[3] de la Torre JC (1994). Impaired brain microcirculation may trigger Alzheimer’s disease. Neurosci Biobehav Rev.

[4] Duncombe J, Kitamura A, Hase Y, Ihara M, Kalaria RN, Horsburgh K (2017). Chronic cerebral hypoperfusion: a key mechanism leading to vascular cognitive impairment and dementia. Closing the translational gap between rodent models and human vascular cognitive impairment and dementia. Clin Sci (Lond).

[5] Ai J, Sun LH, Che H, Zhang R, Zhang TZ, Wu WC (2013). MicroRNA-195 protects against dementia induced by chronic brain hypoperfusion via its anti-amyloidogenic effect in rats. J Neurosci.

[6] Sun LH, Ban T, Liu CD, Chen QX, Wang X, Yan ML (2015). Activation of Cdk5/p25 and tau phosphorylation following chronic brain hypoperfusion in rats involves microRNA-195 down-regulation. J Neurochem.

[7] Chen X, Jiang XM, Zhao LJ, Sun LL, Yan ML, Tian Y (2017). MicroRNA-195 prevents dendritic degeneration and neuron death in rats following chronic brain hypoperfusion. Cell Death Dis.

[8] Cechetti F, Pagnussat AS, Worm PV, Elsner VR, Ben J (2012). da Costa, et al. Chronic brain hypoperfusion causes early glial activation and neuronal death, and subsequent long-term memory impairment. Brain Res Bull.

[9] Heppner FL, Ransohoff RM, Becher B (2015). Immune attack: the role of inflammation in Alzheimer disease. Nat Rev Neurosci.

[10] Hanisch UK, Kettenmann H (2007). Microglia: active sensor and versatile effector cells in the normal and pathologic brain. Nat Neurosci.

[11] David S, Kroner A (2011). Repertoire of microglial and macrophage responses after spinal cord injury. Nat Rev Neurosci.

[12] Ding AH, Nathan CF, Stuehr DJ (1988). Release of reactive nitrogen intermediates and reactive oxygen intermediates from mouse peritoneal macrophages. Comparison of activating cytokines and evidence for independent production. J Immunol.

[13] Mosser DM, Edwards JP (2008). Exploring the full spectrum of macrophage activation. Nat Rev Immunol.

[14] Hu X, Li P, Guo Y, Wang H, Leak RK, Chen S (2012). Microglia/macrophage polarization dynamics reveal novel mechanism of injury expansion after focal cerebral ischemia. Stroke.

[15] Hu X, Leak RK, Shi Y, Suenaga J, Gao Y, Zheng P (2015). Microglial and macrophage polarization-new prospects for brain repair. Nat Rev Neurol.

[16] Colton CA (2009). Heterogeneity of microglial activation in the innate immune response in the brain. J Neuroimmune Pharmacol.

[17] Gordon S (2003). Alternative activation of macrophages. Nat Rev Immunol.

[18] Xiong XY, Liu L, Yang QW (2016). Functions and mechanisms of microglia/macrophages in neuroinflammation and neurogenesis after stroke. Prog Neurobiol.

[19] Kumar A, Alvarez-Croda DM, Stoica BA, Faden AI, Loane DJ (2016). Microglial/macrophage polarization dynamics following traumatic brain injury. J Neurotrauma.

[20] Wang G, Zhang J, Hu X, Zhang L, Mao L, Jiang X (2013). Microglia/macrophage polarization dynamics in white matter after traumatic brain injury. J Cereb Blood Flow Metab.

[21] Zhang L, Xu J, Gao J, Wu Y, Yin M, Zhao W (2018). CD200-, CX3CL1-, and TREM2-mediated neuron-microglia interactions and their involvements in Alzheimer’s disease. Rev Neurosci.

[22] Zujovic V, Benavides J, Vige X, Carter C, Taupin V (2000). Fractalkine modulates TNF-alpha secretion and neurotoxicity induced by microglial activation. Glia.

[23] Kim TS, Lim HK, Lee JY, Kim DJ, Park S, Lee C (2008). Changes in the levels of plasma soluble fractalkine in patients with mild cognitive impairment and Alzheimer’s disease. Neurosci Lett.

[24] Strobel S, Grunblatt E, Riederer P, Heinsen H, Arzberger T, Al-Sarraj S (2015). Changes in the expression of genes related to neuroinflammation over the course of sporadic Alzheimer’s disease progression: CX3CL1, TREM2, and PPARgamma. J Neural Transm (Vienna).

[25] Tang Z, Gan Y, Liu Q, Yin JX, Liu Q, Shi J (2014). CX3CR1 deficiency suppresses activation and neurotoxicity of microglia/macrophage in experimental ischemic stroke. J Neuroinflammation.

[26] Lee S, Varvel NH, Konerth ME, Xu G, Cardona AE, Ransohoff RM (2010). CX3CR1 deficiency alters microglial activation and reduces beta-amyloid deposition in two Alzheimer’s disease mouse models. Am J Pathol.

[27] Yang G, Liu Z, Wang L, Chen X, Wang X, Dong Q (2018). MicroRNA-195 protection against focal cerebral ischemia by targeting CX3CR1. J Neurosurg.

[28] Ding J, Huang S, Wang Y, Tian Q, Zha R, Shi H (2013). Genome-wide screening reveals that miR-195 targets the TNF-alpha/NF-kappaB pathway by down-regulating IkappaB kinase alpha and TAB3 in hepatocellular carcinoma. Hepatology.

[29] Bras JP, Silva AM, Calin GA, Barbosa MA, Santos SG, Almeida MI (2017). MiR-195 inhibits macrophages pro-inflammatory profile and impacts the crosstalk with smooth muscle cells. PLoS One.

[30] Yan M, Ai J (2018). A rodent model for chronic brain hypoperfusion related diseases: permanent bilateral occlusion of the common carotid arteries (2VO) in rats. Bio-protocol.

[31] Sun LL, Duan MJ, Ma JC, Xu L, Mao M, Biddyut D (2018). Myocardial infarction-induced hippocampal microtubule damage by cardiac originating microRNA-1 in mice. J Mol Cell Cardiol.

[32] Theriault P, Bordeleau M, Rivest S (2016). Isolation and purification of murine microglial cells for flow cytometry. Bio-protocol.

[33] Wohleb ES, Hanke ML, Corona AW, Powell ND, Stiner LM, Bailey MT (2011). beta-Adrenergic receptor antagonism prevents anxiety-like behavior and microglial reactivity induced by repeated social defeat. J Neurosci.

[34] Gordon S, Taylor PR (2005). Monocyte and macrophage heterogeneity. Nat Rev Immunol.

[35] Kroner A, Greenhalgh AD, Zarruk JG, Passos Dos Santos R, Gaestel M, David S (2014). TNF and increased intracellular iron alter macrophage polarization to a detrimental M1 phenotype in the injured spinal cord. Neuron.

[36] Yao H, Ma R, Yang L, Hu G, Chen X, Duan M (2014). MiR-9 promotes microglial activation by targeting MCPIP1. Nat Commun.

[37] Moore KJ, Sheedy FJ, Fisher EA (2013). Macrophages in atherosclerosis: a dynamic balance. Nat Rev Immunol.

[38] Goerdt S, Politz O, Schledzewski K, Birk R, Gratchev A, Guillot P (1999). Alternative versus classical activation of macrophages. Pathobiology.

[39] Orihuela R, McPherson CA, Harry GJ (2016). Microglial M1/M2 polarization and metabolic states. Br J Pharmacol.

[40] Mecca C, Giambanco I, Donato R, Arcuri C. Microglia and aging: the role of the TREM2-DAP12 and CX3CL1-CX3CR1 axes. Int J Mol Sci. 2018;19(1):318. 10.3390/ijms19010318 PMC5796261 29361745

[41] Liu YZ, Wang C, Wang Q, Lin YZ, Ge YS, Li DM (2017). Role of fractalkine/CX3CR1 signaling pathway in the recovery of neurological function after early ischemic stroke in a rat model. Life Sci.

[42] Liu Y, Wu XM, Luo QQ, Huang S, Yang QW, Wang FX (2015). CX3CL1/CX3CR1-mediated microglia activation plays a detrimental role in ischemic mice brain via p38MAPK/PKC pathway. J Cereb Blood Flow Metab.

[43] Lauro C, Catalano M, Trettel F, Limatola C (2015). Fractalkine in the nervous system: neuroprotective or neurotoxic molecule?. Ann N Y Acad Sci.

[44] Harrison JK, Jiang Y, Chen S, Xia Y, Maciejewski D, McNamara R (1998). Role for neuronally derived fractalkine in mediating interactions between neurons and CX3CR1-expressing microglia. PNAS.

[45] Nishiyori A, Minami M, Ohtani Y, Takami S, Yamamoto J, Kawaguchi N (1998). Localization of fractalkine and CX3CR1 mRNAs in rat brain: does fractalkine play a role in signaling from neuron to microglia?. FEBS Lett.

[46] Chhor V, Charpentier TL, Lebon S, Oré MV, Celador IL, Josserand VD (2013). Characterization of phenotype markers and neuronotoxic potential of polarised primary microglia in vitro. Brain Behav Immun.

[47] Korzhevskii DE, Kirik OV (2016). Brain microglia and microglial markers. Neurosci Behav Physiol.

[48] Liu CD, Wang Q, Zong DK, Pei SC, Yan Y, Yan ML (2016). Knockdown of microRNA-195 contributes to protein phosphatase-2A inactivation in rats with chronic brain hypoperfusion. Neurobiol Aging.

[49] Gonsalves C, Kalra VK (2010). Endothelin-1-induced macrophage inflammatory protein-1beta expression in monocytic cells involves hypoxia-inducible factor-1alpha and AP-1 and is negatively regulated by microRNA-195. J Immunol.

[50] Grosse GM, Tryc AB, Dirks M, Schuppner R, Pflugrad H, Lichtinghagen R (2014). The temporal dynamics of plasma fractalkine levels in ischemic stroke: association with clinical severity and outcome. J Neuroinflammation.

[51] Soriano SG, Amaravadi LS, Wang YF, Zhou H, Yu GX, Tonra JR (2002). Mice deficient in fractalkine are less susceptible to cerebral ischemia-reperfusion injury. J Neuroimmunol.

