# Supplementary material for: MicroRNA-195 prevents hippocampal microglial/macrophage polarization towards the M1 phenotype induced by chronic brain hypoperfusion through regulating CX3CL1/CX3CR1 signaling
Source: J Neuroinflammation. 2020 Aug 20;17:244. doi: 10.1186/s12974-020-01919-w (PMC7439693; doi:10.1186/s12974-020-01919-w)
Supplement: Supplementary file 1 — Additional file 1: Table S1. Animal Groups and Number of Rats Used in the Study. [file 12974_2020_1919_MOESM1_ESM.docx]

| **Groups** | **Mortality** | **Exclude(dead)** |
| --- | --- | --- |
| **Experiment 1: Time course and IF** |  |  |
| Sham (n=12) | 0 (0/12) | 0 |
| 2VO (1w, 2w, 4w, 8w) | 0 (0/12) | 0 |
| **Experiment 2: Flow cytometry** |  |  |
| Sham (n=3) | 0 (0/3) | 0 |
| 2VO (n=3) | 0 (0/3) | 0 |
| **Experiment 3: Mechanism study** |  |  |
| Sham (n=15) | 0 (0/15) | 0 |
| Negative control (n=9) | 0 (0/9) | 0 |
| Sham+Lenti-AMO*-*195 (n=10) | 10% (1/10) | 1 |
| Sham+Lenti-pre-*miR*-*195*+ Lenti-AMO*-*195 (n=10) | 10% (1/10) | 1 |
| 2VO (n=15) | 6.67% (1/15) | 1 |
| 2VO+ Lenti-pre-*miR*-*195* (n=9) | 0 (0/10) | 0 |
| **TOTAL** |  |  |
| Sham | 0 (0/30) | 0 |
| Model | 5% (3/69) | 3 |

**Table S1 Animal Groups and Number of Rats Used in the Study**

A total of 99 rats were used: 30 in sham group, 69 in model group. Additionally, 3 rats were dead due to 2VO surgery and brain injections.

In **Experiment 3,**  18 rats were used for IF test: 3 in sham group, 3 in NC group, 3 in Sham+Lenti-AMO*-*195 group, 3 in Sham+Lenti-pre-*miR*-*195*+ Lenti-AMO*-*195 group, 3 in 2VO group and 3 in 2VO+ Lenti-pre-*miR*-*195* group. The rest of the rats were used for western blot and PCR test.
