# Supplementary material for: MicroRNA-195 prevents hippocampal microglial/macrophage polarization towards the M1 phenotype induced by chronic brain hypoperfusion through regulating CX3CL1/CX3CR1 signaling
Source: J Neuroinflammation. 2020 Aug 20;17:244. doi: 10.1186/s12974-020-01919-w (PMC7439693; doi:10.1186/s12974-020-01919-w)
Supplement: Supplementary file 2 — Additional file 2: Supplementary Fig. S1. The gating strategy of microglia. Debris and aggregates were eliminated from the analysis by forward- and side-scatter characteristics (small plots). The alive myeloid cells were further identified by CD11b and CD45. Microglia were sorted using CD11b+/ CD45low as a marker. [file 12974_2020_1919_MOESM2_ESM.docx]

**
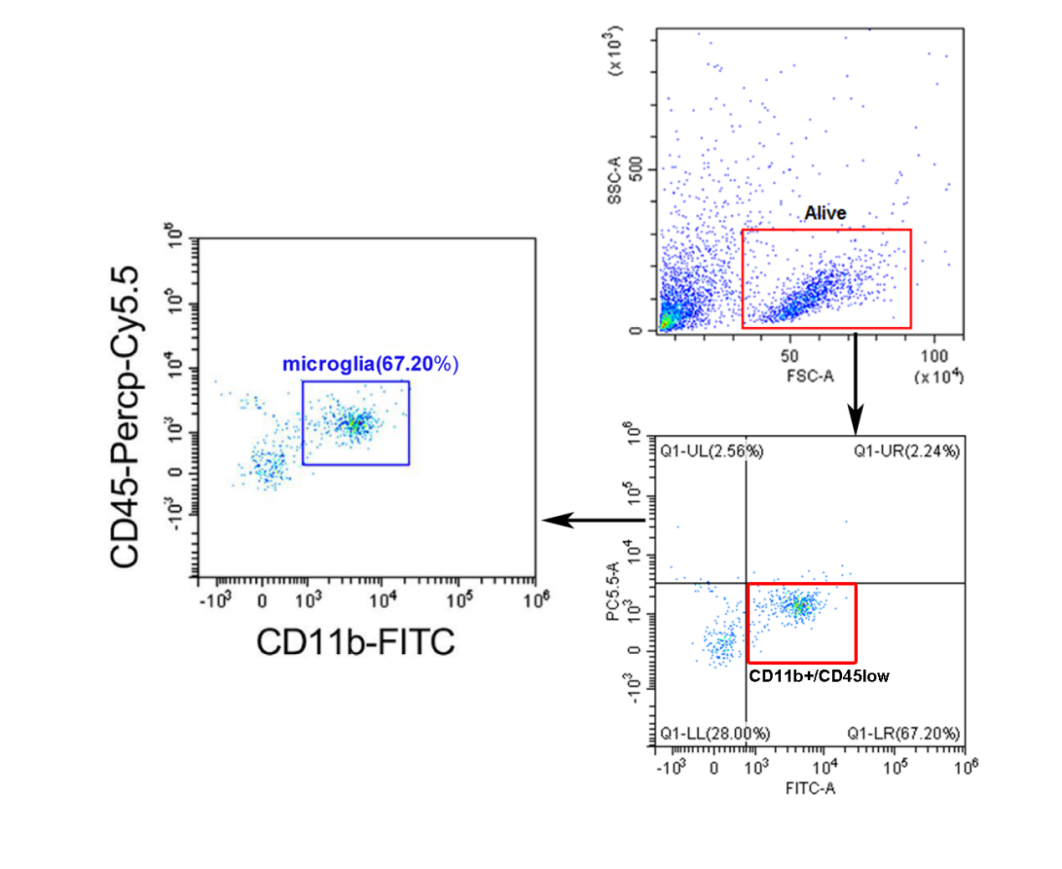
**

**Supplementary Fig.1. The gating strategy of microglia.**

Debris and aggregates were eliminated from the analysis by forward- and side-scatter characteristics (small plots). The alive myeloid cells were further identified by CD11b and CD45. Microglia were sorted using CD11b^+^/ CD45^low^ as a marker.
