# Supplementary material for: MicroRNA-195 prevents hippocampal microglial/macrophage polarization towards the M1 phenotype induced by chronic brain hypoperfusion through regulating CX3CL1/CX3CR1 signaling
Source: J Neuroinflammation. 2020 Aug 20;17:244. doi: 10.1186/s12974-020-01919-w (PMC7439693; doi:10.1186/s12974-020-01919-w)
Supplement: Supplementary file 3 — Additional file 3: Supplementary Fig. S2. MiR-195 prevents LPS induced activation of microglia towards M1 profile of cultured BV2 cells. A. Representative images of CD68 or CD206 expression in Iba-1+BV2 cells by immunofluorescence staining after transfection of NC, miR-195, AMO-195, miR-195+AMO-195, LPS or LPS+miR-195.Scale bar: 40 μm. B&C. Quantification of the percentage of CD68 (B) or CD206 (C) in Iba-1+ BV2 cells. D. Quantification of the ratio of CD68/CD206 in BV2 cells. Bars represent the mean ± SD.; n= 9 from 3 batches of cell culture. *P<0.05 vs NC; #P<0.05 vs AMO-195; $P<0.05 vs LPS. All data were analyzed using one-way ANOVA followed by Tukey test. [file 12974_2020_1919_MOESM3_ESM.docx]

**
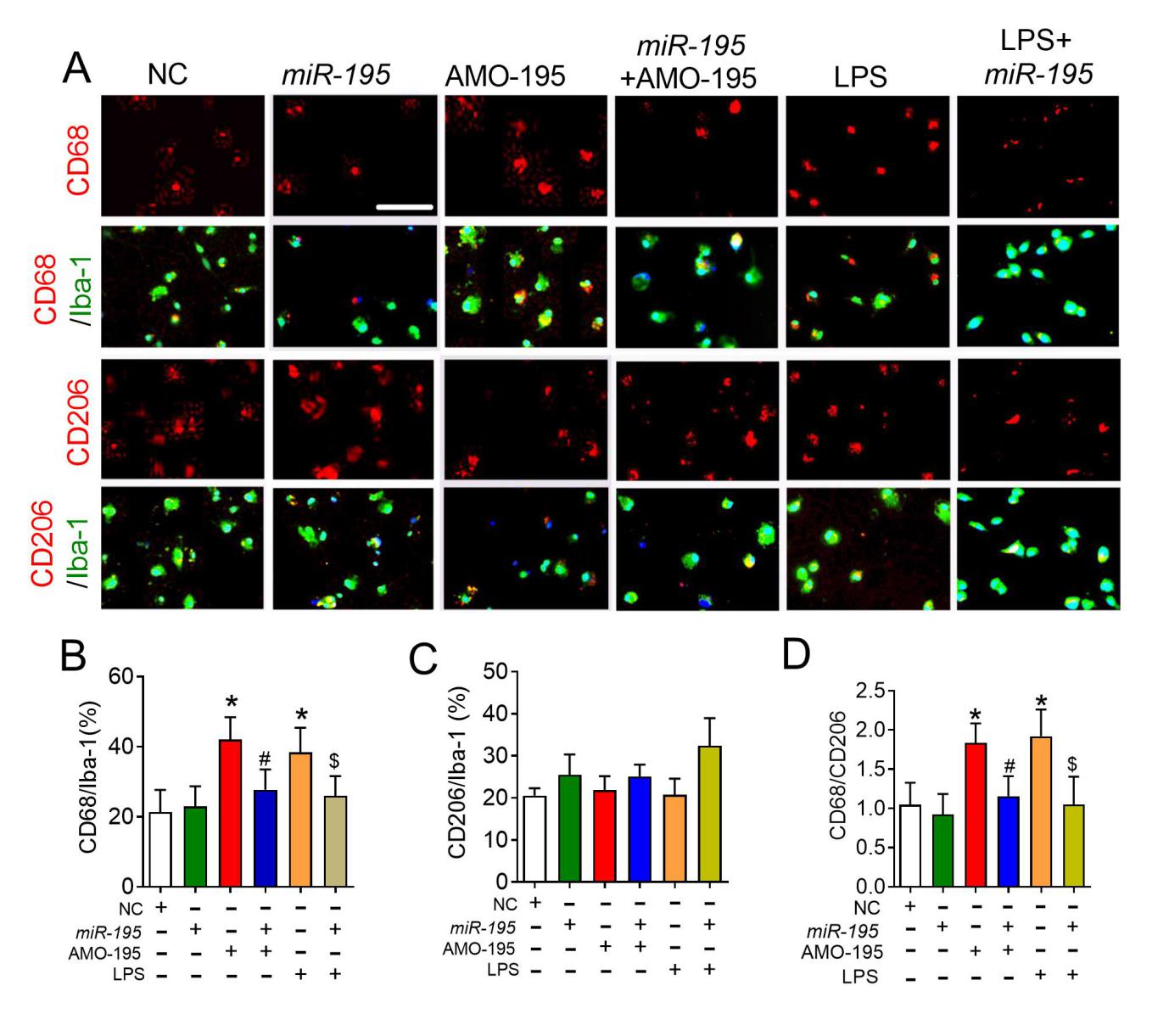
**

**Supplementary Fig.2.** ***MiR-195* prevents LPS induced activation of microglia towards M1 profile of cultured BV2 cells.**

**(A)** Representative images of CD68 or CD206 expression in Iba-1^+^ BV2 cells by immunofluorescence staining after transfection of NC, *miR-195*, AMO-195, *miR-195*+AMO-195, LPS or LPS+*miR-195*.Scale bar: 40 μm. **(B&C)** Quantification of the percentage of CD68 **(B)** or CD206 **(C)** in Iba-1^+^ BV2 cells. **(D)** Quantification of the ratio of CD68/CD206 in BV2 cells. Bars represent the mean ± SD; n= 9 from 3 batches of cell culture. **P*<0.05 *vs* NC; ^#^*P*<0.05 *vs* AMO-195; ^$^*P*<0.05 *vs* LPS. All data were analyzed using one-way ANOVA followed by Tukey test.
